# *ZNF292* mutations impair RNA and protein biosynthesis defining an adverse prognostic subset in early-stage chronic lymphocytic leukemia

**DOI:** 10.1186/s40364-026-01005-y

**Published:** 2026-10-01

**Authors:** Alberto Rodríguez-Sánchez, María Alonso-Gago, Claudia Pérez-Carretero, Luis A. Corchete-Sánchez, Cristina Miguel-García, Sandra Santos-Minguez, Ángela Villaverde-Ramiro, José Antonio Queizán-Hernandez, Araceli Rubio, Rocío Benito-Sánchez, Federico Lo Schiavo, Martina Ghetti, Giorgia Simonetti, Ana E. Rodríguez-Vicente, Jesús M. Hernández-Rivas, Miguel Quijada-Álamo

**Affiliations:** 1 Cancer Research Center, University of Salamanca, IBSAL, IBMCC, CSIC, Salamanca, Spain; 2 Department of Hematology, University Hospital of Salamanca, Salamanca, Spain; 3 Translational Research Centre in Onco-hematology, Faculty of Medicine, University of Geneva, and Swiss Cancer Center Leman, Geneva, Switzerland; 4 Massachusetts General Hospital Center for Cancer Research, Charlestown, MA 02129 USA; 5 Harvard Medical School, Boston, MA 02215 USA; 6 Broad Institute of MIT and Harvard, Cambridge, MA 02139 USA; 7 Department of Hematology, Hospital de Segovia, Segovia, Spain; 8 Department of Hematology, Hospital Miguel Servet, Zaragoza, Spain; 9 Biosciences Laboratory, IRCCS Istituto Romagnolo per lo Studio dei Tumori (IRST) “Dino Amadori”, Meldola, FC Italy; 10 Department of Human Anatomy and Histology, Faculty of Medicine, University of Salamanca, Salamanca, Spain; 11 Department of Oncological Sciences, Icahn School of Medicine at Mount Sinai, New York, NY USA

## Abstract

**Background:**

*ZNF292* alterations are recurrently detected in patients with chronic lymphocytic leukemia (CLL), occurring in 3%-6% of patients as mutations and in 2% as 6q deletions. Although preliminary evidence suggested an independent prognostic role for *ZNF292* mutations, their specific clinical relevance in early-stage CLL and the underlying biological mechanisms remain poorly defined.

**Methods:**

488 CLL patients were analyzed using targeted Next-Generation Sequencing (NGS) to characterize the landscape and clinical significance of *ZNF292* mutations. CRISPR/Cas9-genome editing was employed to introduce the most prevalent *ZNF292* variants into CLL cellular models. Their biological and functional implications were evaluated by integrating RNA-sequencing data from these isogenic cellular models with transcriptomic data from primary patient samples, complemented by functional validation and drug response assays.

**Results:**

*ZNF292* mutations were associated with shorter time to first treatment (TTFT; median 25 vs. 52 months, *P* = 0.009) and inferior overall survival (OS, median 93 vs. 148 months, *P* = 0.004). Importantly, when restricting the analyses to early-stage cases, *ZNF292* alterations were independently associated with shorter TTFT and OS, identifying a high-risk subgroup. Mechanistically, transcriptomic analyses revealed that *ZNF292* mutations suppress global protein biosynthesis and modulate cell cycle progression in both primary CLL samples and CRISPR/Cas9-edited cells. Furthermore, *ZNF292* deficiency impaired replication stress responses via defective CHK1 activation, resulting in DNA damage accumulation. Evaluating potential therapeutic vulnerabilities, *ZNF292*-mutated cells exhibited enhanced in vitro sensitivity to ibrutinib and venetoclax.

**Conclusions:**

Our findings propose *ZNF292* alterations as an independent prognostic factor in early-stage CLL and unveil their role in disease progression.

**Supplementary Information:**

The online version contains supplementary material available at https://doi.org/10.1186/s40364-026-01005-y.

## Background

Chronic lymphocytic leukemia (CLL) is characterized by a highly heterogeneous clinical course determined by a complex genomic landscape [1, 2]. Next-generation sequencing (NGS) studies have delineated the mutational landscape of CLL, uncovering recurrent somatic mutations in up to 100 genes [3–5]. However, only a limited subset, such as *SF3B1*, *NOTCH1*, *TP53* or *XPO1*, has well-established prognostic relevance [6–12]. In contrast, the vast majority occur at low incidence (< 5%), constituting a “long tail” of low-frequency mutations whose significance remains poorly defined [5].

Most patients are diagnosed with asymptomatic early-stage CLL (Rai 0–1/Binet A) and do not require immediate therapy [13,14]. Consequently, current management relies on a watch-and-wait strategy, deferring treatment until the development of symptomatic or progressive disease [15, 16]. This clinical scenario underscores the importance of dissecting the mechanisms underlying early progression [17]. Although two prognostic models for early-stage disease have been developed, they do not incorporate mutations [18, 19]. Given the marked molecular heterogeneity of CLL, integrating recurrent genetic alterations may further refine the prediction of time to first treatment (TTFT) [12, 20].

Within the RNA processing pathway, *ZNF292* (Zinc Finger Protein 292) has emerged as a candidate driver gene in several NGS studies [3–5, 21]. Encoding a C2H2-type protein with 15 zinc-fingers, *ZNF292* may function primarily as a transcription factor, with distinct motifs mediating DNA binding (ZF9-11) and putative RNA or protein interactions [22, 23]. In CLL, somatic *ZNF292* mutations occur in 3.3%-6.6% of patients [3–5, 21, 24]. Additionally, *ZNF292* localizes to chromosome 6q14.3, a recurrently deleted region in a subset of patients with 6q deletion (del(6q)), reported in approximately 3% of patients [3, 21, 25–28]. Beyond CLL, *ZNF292* alterations have been detected in solid tumors [29–32] and linked to cell cycle regulation through the SKP2-p27 axis [33].

Recent large-scale analyses identified *ZNF292* mutations among the small subset of genetic lesions independently associated with clinical outcome, suggesting a potential prognostic role [5]. Nevertheless, their clinical impact has not been addressed in independent cohorts, particularly in early-stage CLL, and their functional contribution to CLL pathogenesis remains undefined. In this study, we assess the prognostic significance of *ZNF292* alterations with a specific focus on early-stage disease. In parallel, we generated CRISPR/Cas9-edited isogenic cells and integrated their transcriptomic profiles with RNA-sequencing data from CLL patients to elucidate the underlying molecular mechanisms driving *ZNF292*-mutated CLL pathobiology.

## Methods

### Patients

Our cohort comprised 488 patients with CLL from 16 Spanish centers, diagnosed following the International Workshop on CLL (iwCLL) guidelines [15]. Only pre-treatment samples were considered for NGS mutational analysis. Clinical and biological data were centrally processed at the Molecular Cytogenetics Unit of the Cancer Research Center in Salamanca, Spain. The median follow-up was 74 months (Supplementary Table S1-2). All participants provided written informed consent, and the study was approved by the *Comité Ético de Investigación Clínica* of the *Hospital Universitario de Salamanca*.

### Next-generation sequencing (NGS)

Genomic DNA was extracted from peripheral blood or bone marrow CD19-positive B lymphocytes via magnetically activated cell sorting (MACS). Exonic regions of 54 CLL-relevant genes were captured using the Agilent SureSelect^QXT^ Target Enrichment system (Agilent Technologies, Santa Clara, CA, USA), based on a custom-designed NGS panel previously validated [34, 35] (Supplementary Table S3). Sequencing libraries were prepared with the SureSelect^QXT^ Library Kit and run on an Illumina NextSeq platform (Illumina, San Diego, CA, USA). The median sequencing depth was 600 reads per base, with ≥ 100X coverage achieved in 97% of the targeted regions. Bioinformatic processing and variant calling are detailed in the Supplementary Methods.

### CRISPR/Cas9 genome editing

Single guide RNAs (sgRNAs) targeting exon 8 of *ZNF292* (Supplementary Table S4) were designed using CRISPick (https://portals.broadinstitute.org/gppx/crispick/), cloned into the pLKO5.sgRNA.EFS.GFP vector (Addgene_#57822) and transfected into the PGA1 and HG3 Cas9-expressing CLL cell lines [36]. GFP-positive cells were single-cell sorted, expanded and screened for the presence of mutations. For each genotype (*ZNF292*-wild-type (*ZNF292*^WT^) and *ZNF292*-mutated (*ZNF292*^MUT^)), at least three independently derived single-cell clones were established, expanded and used in all subsequent experiments.

### RNA-sequencing (RNA-seq)

Total RNA (200 ng) isolated from CRISPR/Cas9-edited cells was used for library preparation via the TruSeq Stranded mRNA protocol and subsequently sequenced on an Illumina NextSeq 550 platform. Raw paired-end reads underwent quality control, adapter trimming and length filtering using fastp (v0.23) [37]. Transcript quantification was conducted using Salmon within the RaNA-Seq platform [38, 39], followed by differential gene expression analysis utilizing the edgeR package [40]. Genes with an adjusted *P* < 0.01 were considered significant, and results were interactively visualized using Rvisdiff [41]. For functional interpretation, Gene Set Enrichment analysis (GSEA) was performed in preranked mode.

### SUnSET assay

Global protein synthesis was evaluated using the non-radioactive Surface Sensing of Translation (SUnSET) assay, as previously described [42]. Exponentially growing cells were pulsed with 5 µg/mL puromycin (Sigma-Aldrich, St. Louis, MO, USA) for 2, 4 and 6 h at 37 °C. Translation was halted with ice-cold PBS washes. Following protein extraction, immunoblotting was performed using an anti-puromycin monoclonal antibody (Millipore, Burlington, MA, USA). Signal was detected using the Odyssey Imaging system (LI-COR Biosciences, Lincoln, NE, USA). Puromycin incorporation was quantified with ImageJ (Fiji).

### EdU incorporation assay

DNA synthesis was assessed using the Click-iT™ Plus EdU Alexa Fluor™ 488 Flow Cytometry (Thermo Fisher Scientific, Waltham, MA, USA). Cells were incubated with 10 µM EdU (5-Ethynyl-2-deoxyuridine) for 1 h, processed according to the manufacturer’s instructions and acquired on a BD AccuriC6+™ flow cytometer (BD Biosciences, San Jose, CA, USA). Data were analyzed with FlowJo™ software (v10.9). Three independent experiments were performed.

### EU global RNA synthesis assay

Global nascent RNA synthesis was evaluated using the Click-iT RNA Imaging Kit Alexa Fluor 594 (Invitrogen, Carlsbad, CA, USA; C10330). Briefly, 5 × 10^5^ cells were plated onto poly-L-lysine-coated coverslips (Sigma-Aldrich) and labeled with 1 mM EU (5-ethynyl uridine) for 20 min at 37 °C. Cells were fixed with 4% paraformaldehyde, permeabilized with 0.5% Triton X-100 and subjected to Click-iT reaction. DNA was stained with DAPI and images were acquired with a Leica SP8 confocal microscope (Leica Microsystems, Wetzlar, Germany). Quantification was performed in ImageJ (Fiji) by measuring the integrated density (IntDen) of the Alexa Fluor 594 signal within DAPI-defined nuclear masks. IntDen, calculated as the product of nuclear area and mean fluorescence intensity, was utilized to accurately reflect total RNA synthesis while accounting for potential variations in nuclear size. Fluorescence intensities were quantified per nucleus.

### Statistical analysis

Student’s t-test, Mann-Whitney U test or ANOVA were performed using GraphPad Prism v8.0.1 (GraphPad Software, San Diego, CA, USA). Clinical impact analyses were conducted with IBM SPSS Statistics v23.0 (IBM, Armonk, NY, USA) and R (v4.2.2). Continuous and categorical variables were analyzed using the Mann-Whitney U and chi-square tests, respectively. Time to first treatment (TTFT) and overall survival (OS) were calculated from the date of diagnosis to treatment initiation or death date, respectively. Survival outcomes were compared using the Kaplan-Meier method with the log-rank test. Cox regression was applied for multivariate TTFT and OS analyses. For single-cell imaging-based analyses, individual cells were considered nested observations within independent experiments, acknowledging the non-biological independence of single-cell data points. Statistical significance was set at *P* < 0.05.

### Supplementary methods

Additional details on NGS, cell lines, culture conditions, drugs, reagents, RNA-seq, CLLmap analysis and functional experiments are provided in the Supplementary Methods.

## Results

### *ZNF292* alterations define an independent prognostic subgroup in early-stage CLL

**Fig. 1:**
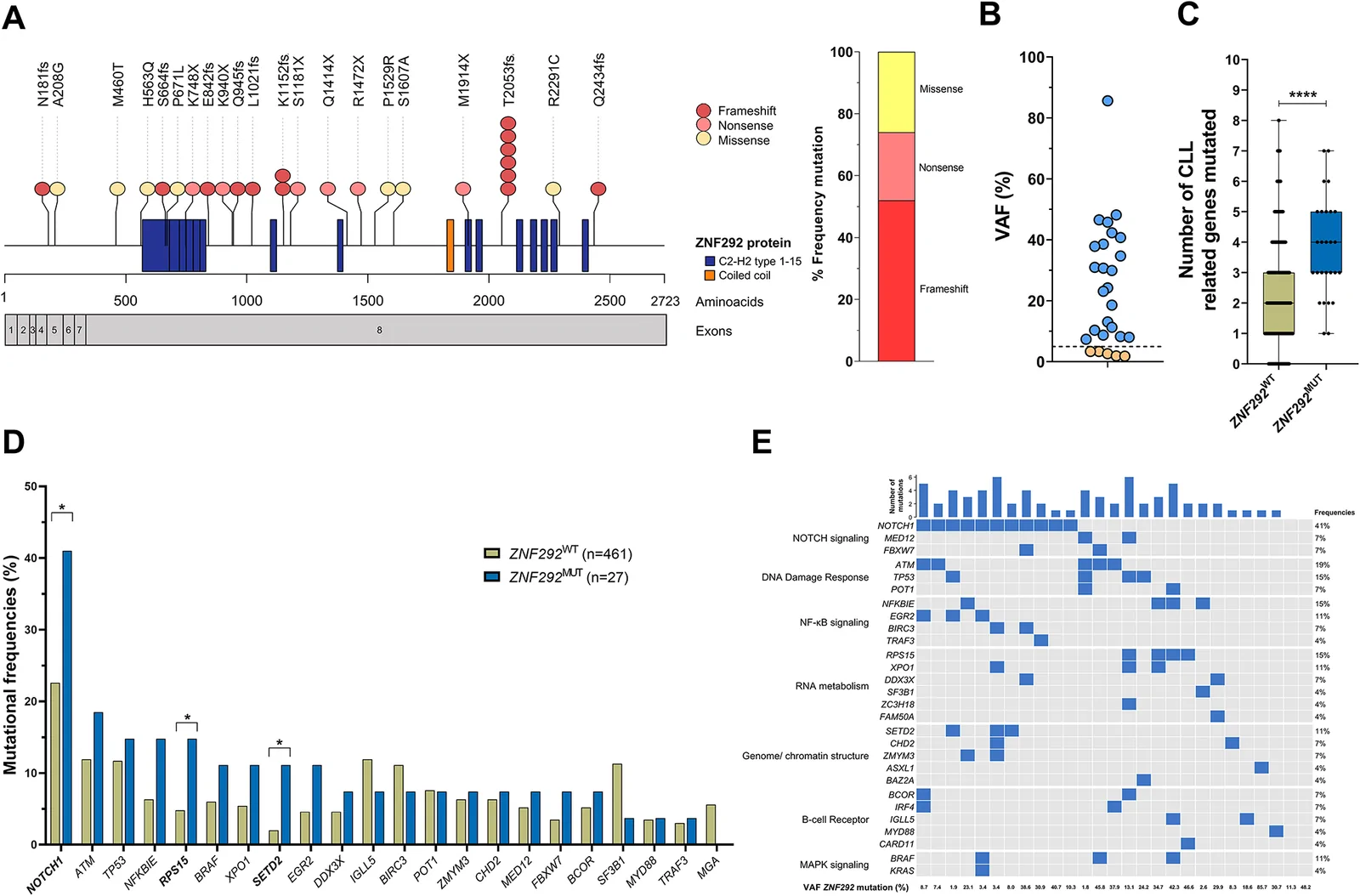
Molecular and mutational landscape of *ZNF292* mutations in CLL patients. **A**. Left. Schematic representation of the ZNF292 protein structure mapping the distribution of the identified mutations. Exon distribution is depicted in the lower grey bar. C2-H2 zinc finger motifs and coiled-coil domains are highlighted in blue and orange, respectively. Color-coded circles indicate the position and type of mutation. Right. Bar chart showing the relative frequencies of frameshift (red), nonsense (light red), and missense (yellow) mutations, highlighting a predominance of truncating events. **B**. Variant allele frequency (VAF, %) distribution for all detected *ZNF292* mutations. The dashed line indicates the 5% VAF threshold. **C**. Boxplot comparing the total mutational burden (number of concurrent mutations in the 54 CLL-related driver genes analyzed) between *ZNF292*^WT^ and *ZNF292*^MUT^ cases. Statistical significance was determined using the Mann-Whitney U test (**** *P* < 0.0001). The boxes represent the median and interquartile ranges, while whiskers indicate the minimum and maximum values. **D**. Bar graph comparing the mutational frequencies (%) of recurrently altered CLL genes between *ZNF292*^WT^ (*n* = 461) and *ZNF292*^MUT^ (*n* = 27) patients. Significant genetic co-occurrences were observed for *NOTCH1*, *RPS15*, and *SETD2* (* *P* < 0.05). **E**. Oncoplot detailing the concurrent mutational landscape of the *ZNF292*^MUT^ cohort. Patients (columns) are annotated for specific genetic alterations (blue squares). Genes (rows) are grouped according to their primary biological pathways. The top bar graph displays the total number of mutations per individual patient, and the right-hand percentages indicate the mutational frequency of each specific gene within this targeted subgroup

A total of twenty-seven *ZNF292* mutations were detected in 27/488 CLL patients (5.5%) (Fig. 1A). Of these variants, 74% were predicted to generate truncated proteins, including frameshift (52%, 14/27) or nonsense (22%, 6/27) events. The remaining 7 missense mutations affected highly conserved residues and were bioinformatically predicted as deleterious (Supplementary Fig. 1A). *ZNF292* mutations were broadly distributed throughout the coding region, with a recurrent frameshift mutation (p.T2053fs) identified in 6 cases. Variant allele frequencies (VAFs) showed a wide distribution, ranging from 1.8% to 85.6% (Fig. 1B), with the highest VAF value corresponding to a patient harboring a concurrent 6q deletion encompassing the *ZNF292* locus. A similar mutational pattern has been reported in an independent CLL cohort [5] (Supplementary Fig. 1B-C). Beyond point mutations, we identified 7 cases in the study cohort harboring a del(6q) encompassing the ZNF292 locus [28]. RNA-seq data analysis of patients from the CLLmap cohort [5] revealed no significant differences in *ZNF292* expression between wild-type and mutated patients (Supplementary Fig. 1D).

*ZNF292* mutations were significantly enriched in IGHV-UM CLL (76.0% vs. 50.6%, *P* = 0.011) and among patients requiring therapy (76.9% vs. 56.2%, *P* = 0.028), with no significant differences observed in other clinicobiological parameters (Supplementary Table S1). Moreover, *ZNF292*-mutated patients displayed a higher mutational burden within the targeted panel of 54 CLL-related genes than *ZNF292*-wildtype cases (Fig. 1C). Assessment of cytogenetic and genetic associations revealed that *ZNF292* mutations were significantly mutually exclusive with 13q deletion (29.2% vs. 49.4%; *P* = 0.042; Supplementary Fig. 1E), and frequently co-occurred with *NOTCH1* (11/27, 40.7%), *RPS15* (4/27, 14.8%) and *SETD2* (3/27, 11.1%) alterations (Fig. 1D-E, Supplementary Table S5). The association between *ZNF292* and *NOTCH1* mutations was further validated in the CLLmap cohort [5] (Supplementary Fig. 2A). VAF analyses in *NOTCH1* co-mutated cases showed that *ZNF292* VAFs were comparable to or lower than those of *NOTCH1* (Supplementary Fig. 2B), suggesting their emergence as late subclonal events relative to *NOTCH1*.

**Fig. 2:**
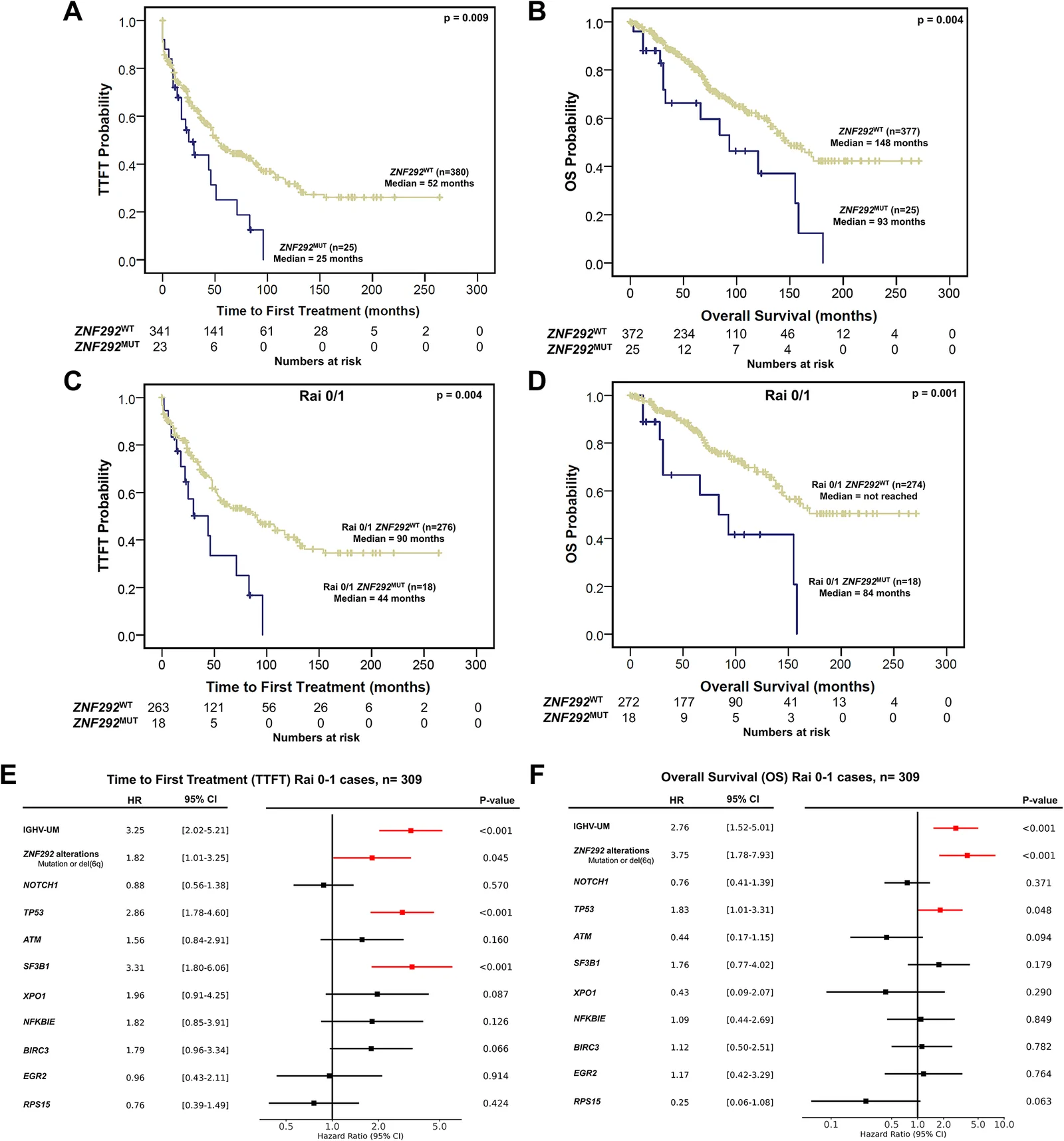
Clinical impact of *ZNF292* alterations in CLL. **A**, **B**. Kaplan-Meier estimates of Time to First Treatment (TTFT) (**A**) and Overall Survival (OS) (**B**) for the entire cohort, stratified by ZNF292 mutational status (ZNF292WT in beige; ZNF292MUT in blue). Median survival times in months and the number of patients (n) per group are indicated. Statistical significance was determined using the log-rank test. **C**, **D**. Subgroup survival analysis evaluating TTFT (**C**) and OS (**D**) exclusively in patients presenting with early-stage disease at diagnosis (Rai stage 0 or 1). **E**, **F**. Forest plots detailing the multivariate Cox proportional hazards regression analysis for TTFT (**E**) and OS (**F**) in Rai 0–1 cases (*n* = 309). The analysis incorporates *ZNF292* alterations (mutation or del(6q)) alongside advanced Rai stage and molecular prognostic factors. Squares represent the Hazard Ratios (HR), and horizontal lines indicate the 95% Confidence Intervals (CI). Variables retaining statistical significance (*P* < 0.05) are highlighted in red

The genomic disruption of *ZNF292* was associated with adverse clinical outcomes. *ZNF292* mutations were associated with a significantly shorter time to first treatment (TTFT) (median 25 vs. 52 months; *P* = 0.009) and inferior overall survival (OS) (median 93 vs. 148 months, *P* = 0.004) (Fig. 2A-B). Similar adverse outcomes were observed in patients with del(6q) encompassing *ZNF292* (*n* = 4) (Supplementary Fig. 2C-F). This prognostic stratification was also present in early-stage disease: among Rai 0–1 patients, *ZNF292* mutations were associated with shorter TTFT (median 44 vs. 90 months; *P* = 0.004) and shortened OS (median 84 months vs. not-reached; *P* = 0.001) (Fig. 2C-D, Supplementary Fig. 2G-H). Furthermore, patients harboring concurrent *ZNF292* and *NOTCH1* mutations exhibited shorter TTFT (median 25 vs. 59 months, *P* = 0.005) than patients without concurrent *NOTCH1* or *ZNF292* alterations (Supplementary Fig. 2I). Evaluation of the CLLmap cohort confirmed the negative impact of both *ZNF292* mutations and diminished *ZNF292* expression on OS, TTFT and failure-free survival (FFS), even within treatment-naïve IGHV-UM cases [5] (Supplementary Fig. 3, Supplementary Table S6). Notably, within the subgroup of patients treated with chemo or chemoimmunotherapy, *ZNF292* mutations were also associated with significantly reduced OS compared to wild-type cases (median OS = 16 vs. 307 months, *P* = 0.013; Supplementary Fig. 3I).

To evaluate the independent prognostic value of *ZNF292* alterations, defined as either a mutation (*n* = 25) or del(6q) encompassing the locus (*n* = 4), we performed multivariate Cox regression. Models were adjusted for established prognostic factors, including IGHV status, Rai stage and recurrent gene mutations previously associated with shorter TTFT [12] and enriched among early-stage cases. *ZNF292* alterations remained independently associated with shorter TTFT and inferior OS (Supplementary Fig. 4). Importantly, when this analysis was restricted to Rai 0–1 cases, *ZNF292* alterations remained independently associated with both earlier treatment requirement (Fig. 2E, HR = 1.82, 95% CI = 1.01–3.25, *P* = 0.045), alongside IGHV-UM, *SF3B1* and *TP53* mutations; and inferior OS (Fig. 2F, HR = 3.75, 95% CI = 1.78–7.93, *P* < 0.001), together with IGHV-UM and *TP53* mutations (Supplementary Table S7). These findings indicate that *ZNF292* alterations represent an independent prognostic factor associated with adverse outcomes within early-stage CLL.

### *ZNF292* mutations rewire transcriptional programs governing cell cycle and mRNA translation

Prompted by the adverse clinical impact associated with *ZNF292* disruption, we sought to investigate its functional contribution to CLL pathobiology. Using CRISPR/Cas9 genome editing, we established isogenic *ZNF292*^MUT^ in 2 CLL-derived cell lines, PGA1 and HG3. Because *ZNF292* variants predominantly cluster within exon 8, we targeted its 5’ region to generate biallelic frameshift mutations, causing premature truncation and loss of all functional domains. Sanger sequencing confirmed the successful introduction of the indels, and immunoblot analysis verified the absence of *ZNF292* protein in the resulting mutated clones (Supplementary Fig. 5).

To delineate the global transcriptional impact associated with *ZNF292* disruption, we performed RNA-seq analyses on *ZNF292*^MUT^ compared with *ZNF292*^WT^ using two isogenic CRISPR/Cas9-edited cell lines: PGA1 (*n* = 4 per genotype) and HG3 (*n* = 3 per genotype). We identified 46 significantly upregulated and 45 significantly downregulated differentially expressed genes (DEGs, |log_2_ fold change [FC]|>0.75, FDR < 0.01) between *ZNF292*^WT^ and *ZNF292*^MUT^ PGA1 cells (Supplementary Fig. 6). Similarly, HG3 *ZNF292*^MUT^ cells exhibited 71 DEGs (5 upregulated and 66 downregulated).

**Fig. 3:**
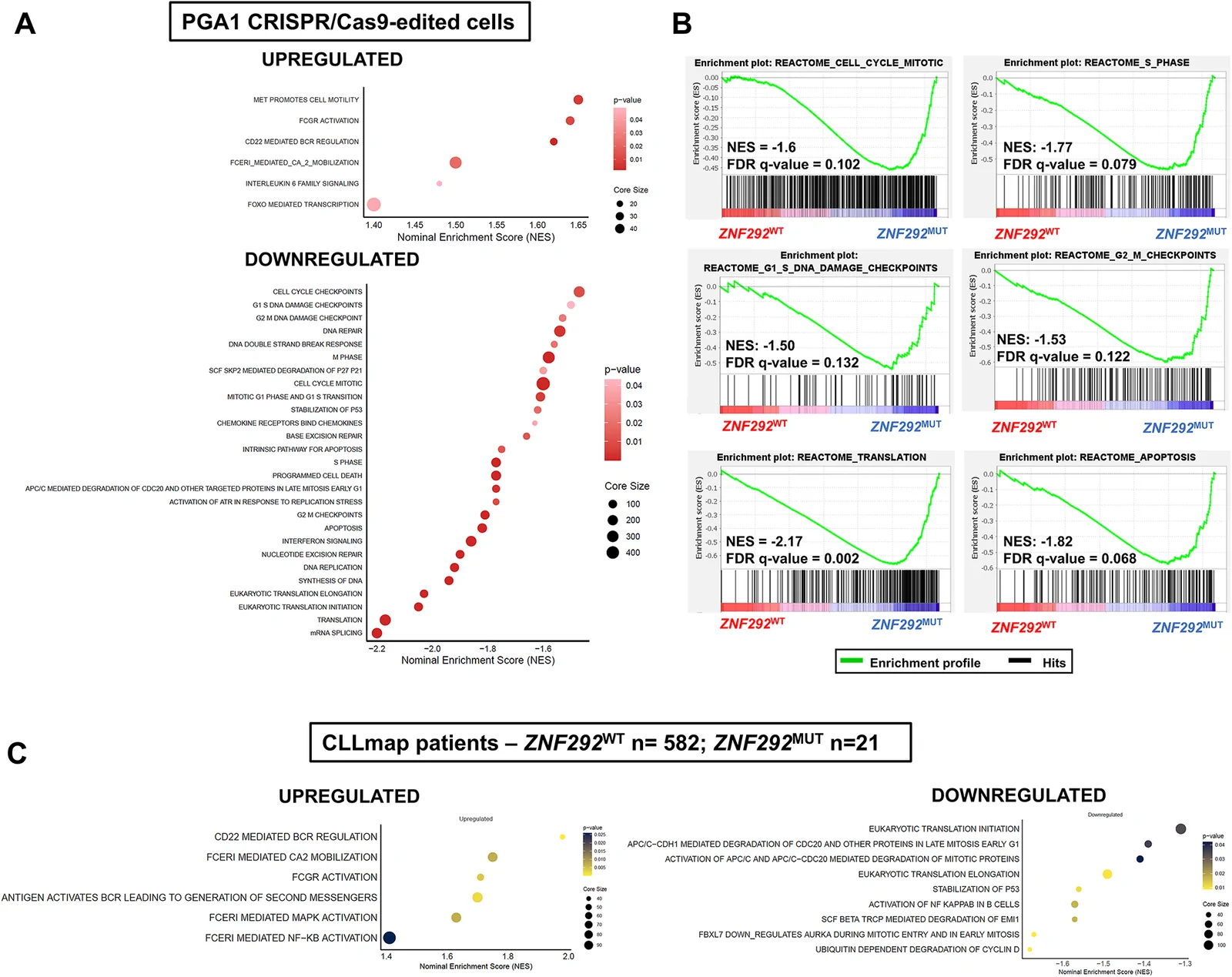
Transcriptomic profiling of *ZNF292*^MUT^ CLL CRISPR/Cas9-edited cells and CLL patients. **A**. GSEA was performed in CRISPR/Cas9-edited *ZNF292*^MUT^ cells compared to *ZNF292*^WT^ PGA1 cells. Dot plots display the top significantly upregulated (top) and downregulated (bottom) functional pathways, ranked by Nominal Enrichment Score (NES). The node size (Core Size) corresponds to the number of genes driving the enrichment, and the color gradient represents the nominal *P*-value. **B**. Representative GSEA enrichment plots detailing the negative enrichment of key biological processes in *ZNF292*^MUT^ PGA1 CRISPR/Cas9-edited cells. Pathways related to cell cycle progression (mitotic, S-phase, and checkpoints), mRNA translation, and apoptosis are significantly downregulated. The green line traces the enrichment profile, while vertical black bars denote individual gene hits within the ranked transcriptomic list. **C**. Transcriptomic signatures in primary patient samples from the CLLmap cohort (*ZNF292*^WT^, *n* = 582; *ZNF292*^MUT^, *n* = 21). GSEA dot plots highlight the significant downregulation of eukaryotic translation and mitotic transition pathways in *ZNF292*-mutated patients

To elucidate the molecular programs dysregulated by *ZNF292* mutations, we conducted pre-ranked GSEA using the Reactome database. In the PGA1 cells, *ZNF292*^MUT^ cells demonstrated a significant upregulation of pathways related to B-cell receptor (BCR) signaling (CD22-mediated BCR regulation), immune modulation (IL-6 signaling) and MET-driven cellular motility (Fig. 3A). Conversely, negatively enriched gene sets were predominantly associated with mRNA splicing and translation, cell cycle progression (S-phase and cell cycle checkpoints), DNA damage repair (double-strand break response, base excision repair), apoptosis, p53 signaling and the replication stress response (Fig. 3B, Supplementary Table S8). Importantly, parallel pathway analysis of the differentially expressed signatures in the HG3 cells corroborated the downregulation of highly overlapping biological processes, mainly impacting cell cycle dynamics and mRNA translation (Supplementary Fig. 7).

To establish the clinical relevance of this *ZNF292*-associated transcriptomic signature, and address the inherent limitations of using Epstein-Barr virus (EBV)-transformed cell lines [43, 44], we interrogated RNA-seq data from CLL patients within the publicly available CLLmap dataset [5] (582 *ZNF292*^WT^, 21 *ZNF292*^MUT^) (Supplementary Table S9). GSEA demonstrated a functional concordance between CRISPR/Cas9-edited models and patient samples (Fig. 3C, Supplementary Fig. 8, Supplementary Table S10). Specifically, BCR signaling pathways were positively enriched in *ZNF292*^MUT^ patients, whereas cell cycle regulation, particularly involving the anaphase-promoting complex (APC), as well as mRNA translation and p53 stabilization, were significantly downregulated in *ZNF292*^MUT^ patients. Moreover, *ZNF292*^MUT^ samples exhibited a significant downregulation of proteostasis components and key regulators of mRNA translation. Conversely, essential G1/S-phase and BCR regulators were upregulated (Supplementary Fig. 9). Together, these findings indicate that the transcriptional programs associated with *ZNF292* mutations predominantly impact BCR signaling, cell cycle regulation and mRNA translation.

### *ZNF292* loss alters S-phase progression and suppresses global biosynthetic activity

**Fig. 4:**
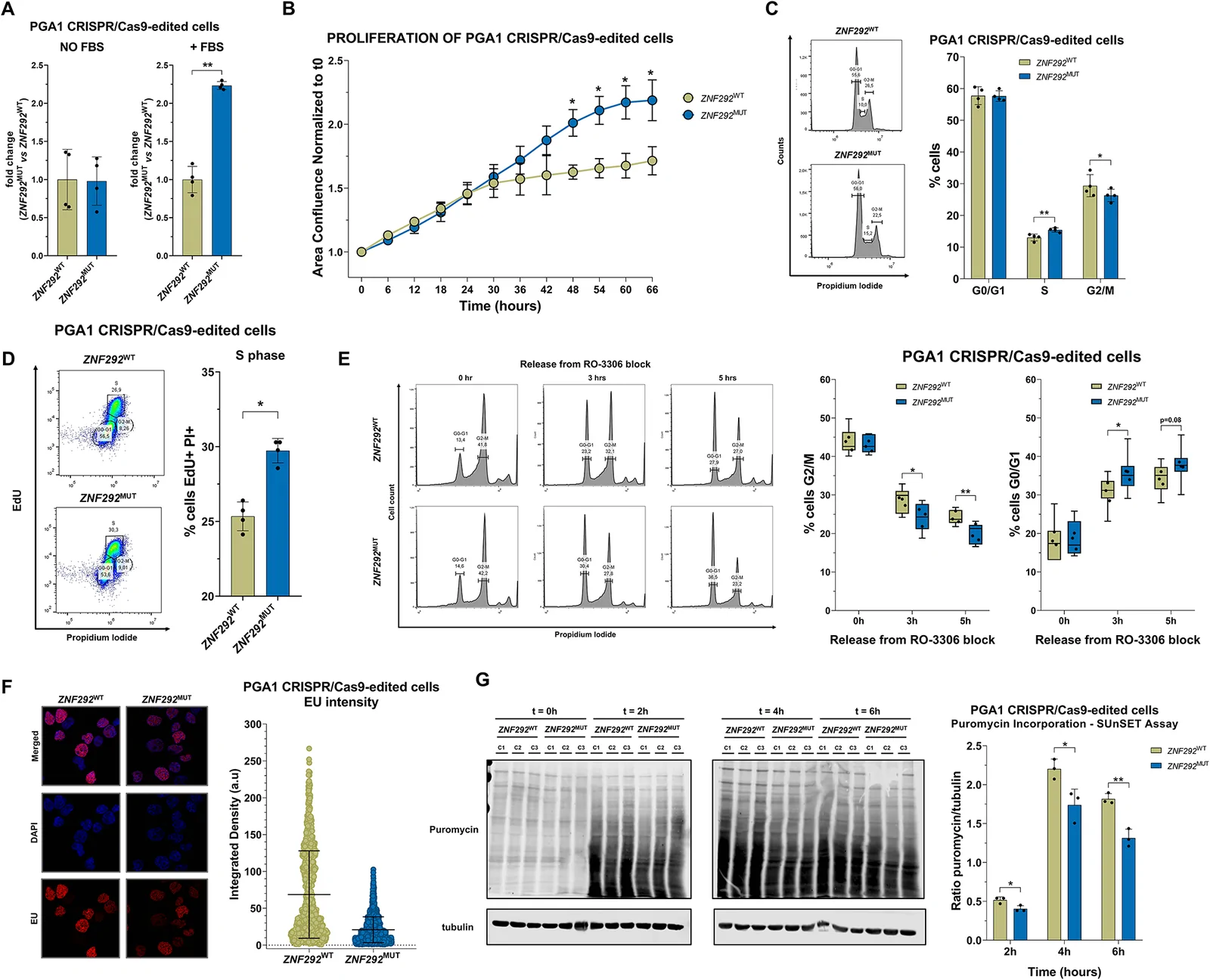
*ZNF292* mutations extend S phase and reduce global RNA and protein synthesis in CLL cells. **A**. Transwell migration assay of *ZNF292*^WT^ and *ZNF292*^MUT^ PGA1 CRISPR/Cas9-edited cells. Bar graphs represent the fold-change in migration under basal conditions (without fetal bovine serum-FBS) and in response to a chemoattractant (+ FBS). Data represent mean ± SD of 3 independent biological clones per genotype; technical duplicates were averaged per clone. Statistical significance was evaluated using a two-tailed unpaired Student’s t-test performed independently for each condition (** *P* < 0.01). **B**. Real-time kinetic proliferation assay using the IncuCyte system. Cellular growth of PGA1 isogenic cells is expressed as phase-contrast area confluence normalized to the initial time point (t = 0) over a 66-hour period. Data points represent mean ± SD of 4 independent biological clones per genotype evaluated across 2 independent experiments. Statistical differences were analyzed using a Two-Way ANOVA (* *P* < 0.05). **C**. Flow cytometric cell cycle analysis using Propidium Iodide (PI) staining in PGA1 CRISPR/Cas9-edited cells. Left: Representative histograms displaying cell cycle phase distribution. Right: Quantitative bar chart showing cell cycle phase percentages. Individual data points represent the mean value of 4 independent biological clones evaluated across 3 independent experiments. Statistical comparisons across cell cycle phases (G0/G1, S, and G2/M) were performed using a Two-Way ANOVA (* *P* < 0.05; ** *P* < 0.01). **D**. Flow cytometric quantification of active DNA synthesis via EdU pulse-labeling in PGA1 CRISPR/Cas9-edited cells. Left: Representative biaxial plots (PI vs. EdU). Right: Bar graph showing the percentage of EdU + PI+ cells. Individual data points represent 4 independent biological clones evaluated across 3 independent experiments (data presented as mean ± SD). Genotypes were compared using a two-tailed unpaired Student’s t-test (* *P* < 0.05). **E**. Left. Flow cytometric analysis of G2/M synchronized (RO-3306 for 20 h) in PGA1 CRISPR/Cas9-edited cells. Representative histograms show an accelerated exit from G2/M into the G1 phase after release in full medium. Right. Box plot showing percentages of cells in G2/M and G0/G1 that were released from RO-3306 synchronization (n = 3 independent experiments). Boxes display medians with 25th to 75th percentiles and whiskers min-max values, with individual data points representing independent biological clones (4 per genotype). Significances were estimated using a Two-Way ANOVA (* P < 0.05; ** P < 0.01). **F**. Confocal immunofluorescence microscopy of nascent RNA synthesis using 5-ethynyl uridine (EU) incorporation in *ZNF292*^WT^ (*n* = 3) and *ZNF292*^MUT^ (*n* = 3) PGA1 CRISPR/Cas9-edited cells. Left: Representative merged, DAPI (nuclei, blue), and EU (red) images. Right: Violin plot of the integrated density showing an impairment of global transcription in *ZNF292*^MUT^ PGA1 cells. Each dot represents an individual cell nucleus. Statistical significance was calculated using the Mann-Whitney U test (*P* < 0.0001). **G**. Evaluation of global protein synthesis rates via Surface Sensing of Translation (SUnSET) assay in PGA1 CRISPR/Cas9-edited cells. Left: Representative Western blot of puromycin incorporation into nascent peptides at 0, 2, 4, and 6 h. β-tubulin was used as a loading control. Right: Densitometric quantification (puromycin/tubulin ratio). Data represent mean ± SD of 3 independent biological clones evaluated across 3 independent experiments. Differences across time points were analyzed using a Two-Way ANOVA (* P < 0.05; ** P < 0.01)

Guided by the transcriptomic findings, we functionally evaluated the implications of *ZNF292* mutations on cell motility, cell cycle progression, and global biosynthetic dynamics. In Transwell assays using fetal bovine serum (FBS) as chemoattractant, *ZNF292*^MUT^ cells exhibited a significantly enhanced migratory capacity, evidenced by an approximately two-fold increase in migrating cells compared to *ZNF292*^WT^ cells (Fig. 4A). Moreover, *ZNF292*^MUT^ cells showed higher growth rates than their wild-type counterparts (Fig. 4B).

Next, we investigated whether the transcriptomic downregulation of cell cycle programs translated into altered cell cycle dynamics. DNA-content analysis revealed a modest but significantly higher proportion of *ZNF292*^MUT^ cells in S phase, accompanied by a reduction in the G2/mitosis fraction (Fig. 4C). This altered profile was validated in the HG3 isogenic cells (Supplementary Fig. 10A). Cell cycle profiling following a short EdU pulse further demonstrated that *ZNF292*^MUT^ cells have an extended S-phase, measured by the accumulation of EdU-positive cells (Fig. 4D). Investigating the impact of *ZNF292* on mitotic progression, *ZNF292*^MUT^ cells had transitioned faster through mitosis within 3 to 5 h after release from G2 synchronization (Fig. 4E). Given the transcriptomic dysregulation of the p27-SKP2 regulatory axis in *ZNF292*^MUT^ cells [33], we examined the expression of p27, SKP2 and c-MYC. Immunoblotting revealed p27 upregulation and reduced c-MYC levels in *ZNF292*^MUT^ cells, whereas SKP2 expression remained unchanged. Notably, an inverse correlation between *ZNF292* and p27 (*CDKN1B*) was also observed in patients from CLLmap (Supplementary Fig. 10B-D).

Considering the proposed function for *ZNF292* in transcriptional regulation, we explored its impact on transcription by quantifying nascent RNA synthesis at the single-cell level using 5-ethynyl uridine (EU) pulse-labeling. Under basal conditions, *ZNF292*^MUT^ cells exhibited reduced EU incorporation compared to *ZNF292*^WT^ (Fig. 4F; Mann-Whitney U test, *P* < 0.0001, statistical comparison based on individual-cell measurements from three independent clones per genotype). This defect was accompanied by an attenuation in protein synthesis efficiency. Using the SUnSET assay, we observed a pronounced decrease in puromycin-labeled polypeptides in *ZNF292*^MUT^ cells at all evaluated time points (Fig. 4G). Collectively, these functional assays corroborate the transcriptomic findings of downregulated translation-related pathways, demonstrating that *ZNF292* mutations suppress RNA and protein biosynthesis.

### *ZNF292* mutations disrupt replication stress response through impaired ATR-CHK1 signaling

**Fig. 5:**
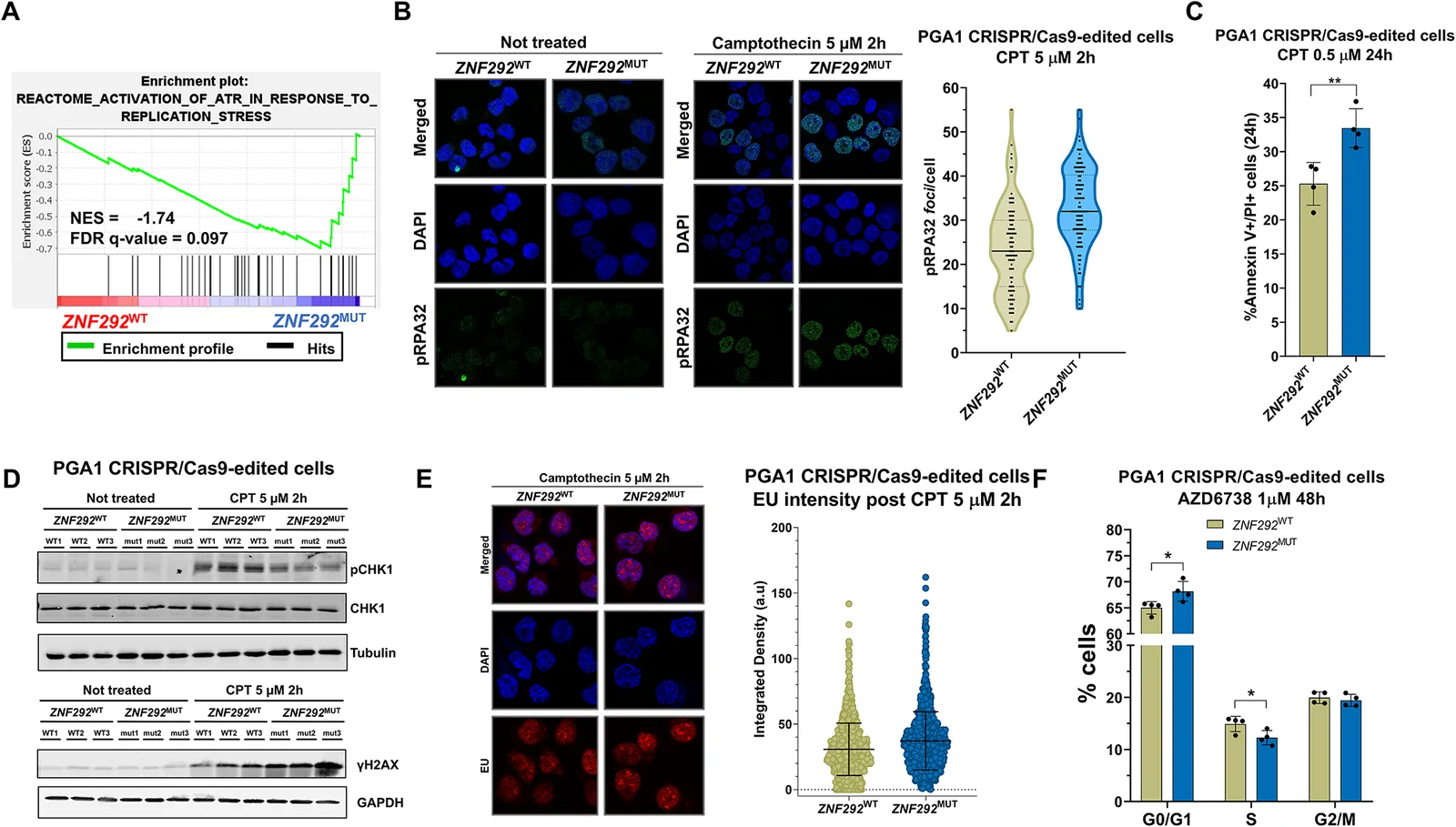
Impact of *ZNF292* mutations in response to replication stress. **A**. GSEA plot showing a significant negative enrichment of the “Activation of ATR in response to replication stress” Reactome pathway in ZNF292MUT PGA1 CRISPR/Cas9-edited cells compared to ZNF292WT. **B**. Confocal immunofluorescence analysis of phosphorylated RPA32 (pRPA32) nuclear foci following induction of replication stress with camptothecin (CPT; 5 µM for 2 h) in PGA1 CRISPR/Cas9-edited cells. Left: representative confocal images. Right: Violin plots showing pRPA32 foci counts per nucleus in the pRPA32-positive subpopulation. Each dot represents an individual cell nucleus pooled from 3 independent biological clones per genotype. Statistical significance was evaluated using the Mann-Witney U (P < 0.0001). **C**. Flow cytometric quantification of apoptosis (Annexin V+/PI+ cells) following a 24-hour treatment with CPT (5 µM) in PGA1 CRISPR/Cas9-edited cells. Data depict mean ± SD, with individual data points representing 3 independent biological clones evaluated across 3 independent experiments. Statistical comparisons were performed using a two-tailed unpaired Student’s t-test (** *P* < 0.01). **D**. Western blot analysis of CHK1 and phospho-CHK1 under basal conditions (not treated) and after CPT exposure (5 µM, 2 h) in PGA1 CRISPR/Cas9-edited cells. Tubulin and GAPDH were used as loading controls. **E**. Assessment of 5-ethynyl uridine (EU) incorporation after CPT treatment (5 µM, 2 h) in PGA1 CRISPR/Cas9-edited cells. Violin plots show single-cell nuclear integrated density values, where each dot represents an individual cell nucleus pooled across 3 biological clones per genotype. Statistical comparison was performed using the Mann-Whitney U test (P < 0.0001). **F**. Cell cycle phase distribution evaluated by flow cytometry after selective pharmacological inhibition of ATR using AZD6738 (1 µM for 48 h) in PGA1 CRISPR/Cas9-edited cells. Bar graphs display mean ± SD, with individual points representing 4 independent biological clones evaluated across 3 independent experiments. Differences between genotypes across cell cycle phases were analyzed using a Two-Way ANOVA (* P < 0.05)

Based on the transcriptional downregulation of DNA damage repair and replication stress response pathways in *ZNF292*^MUT^ cells (Figs. 3A and 5A), we investigated whether *ZNF292* disruption compromises the response to replication-associated DNA damage. To this end, we treated PGA1 cells with camptothecin (CPT), a topoisomerase I (TOP1) inhibitor that induces replication-associated DNA double-strand breaks during S phase. Following CPT exposure, *ZNF292*^MUT^ cells exhibited a significantly higher accumulation of phosphorylated RPA32 (p-RPA32), a marker for DNA end resection and single-strand DNA generation, indicating that *ZNF292*^MUT^ cells harbor higher replication stress (Fig. 5B; Mann-Whitney U test, *P* < 0.0001, statistical comparison based on individual-cell measurements from three independent clones per genotype). Interestingly, CPT treatment also resulted in increased apoptosis in *ZNF292*^MUT^ cells (Fig. 5C, Supplementary Fig. 11A).

To further delineate the DNA damage response signaling of *ZNF292*^MUT^, we evaluated the ATR-CHK1 axis under replication stress (RS). Immunoblot analysis following CPT revealed a reduction in the phosphorylation of CHK1 (pCHK1), the canonical downstream effector of ATR, in *ZNF292*^MUT^ cells (Fig. 5D), pointing to impaired activation of the ATR-CHK1 checkpoint despite induced DNA damage. Furthermore, *ZNF292*^MUT^ cells exhibited persistent DNA damage, as evidenced by increased γH2AX signaling (Fig. 5D, Supplementary Fig. 11B). Remarkably, while CPT treatment triggered the expected suppression of nascent RNA synthesis in *ZNF292*^WT^ cells, *ZNF292*^MUT^ cells failed to reduce EU incorporation in response to CPT relative to both their basal state and their *ZNF292*^WT^ counterparts (Fig. 5E, Supplementary Fig. 11C; Mann-Whitney U test, *P* < 0.0001, statistical comparison based on individual-cell measurements from three independent clones per genotype), suggesting that *ZNF292*^MUT^ cells do not suppress RNA synthesis in replication stress conditions.

Given the central role of ATR in coordinating S phase progression and resolving replication stress [45], we analyzed cell cycle dynamics following pharmacological ATR inhibition. Upon AZD6738 treatment, both *ZNF292*^WT^ and *ZNF292*^MUT^ cells showed an overall increase in the G0/G1 fraction and a concomitant reduction in G2/M population (Supplementary Fig. 11D). However, ATR blockade exerted opposing effects on S-phase distribution depending on *ZNF292* status: whereas *ZNF292*^WT^ cells accumulated in S phase, *ZNF292*^MUT^ cells displayed a marked reduction in the S-phase fraction (Fig. 5F), indicating that *ZNF292* mutations modulate S-phase progression.

### *ZNF292* mutations confer enhanced sensitivity to ibrutinib, venetoclax and palbociclib in CLL cells

**Fig. 6:**
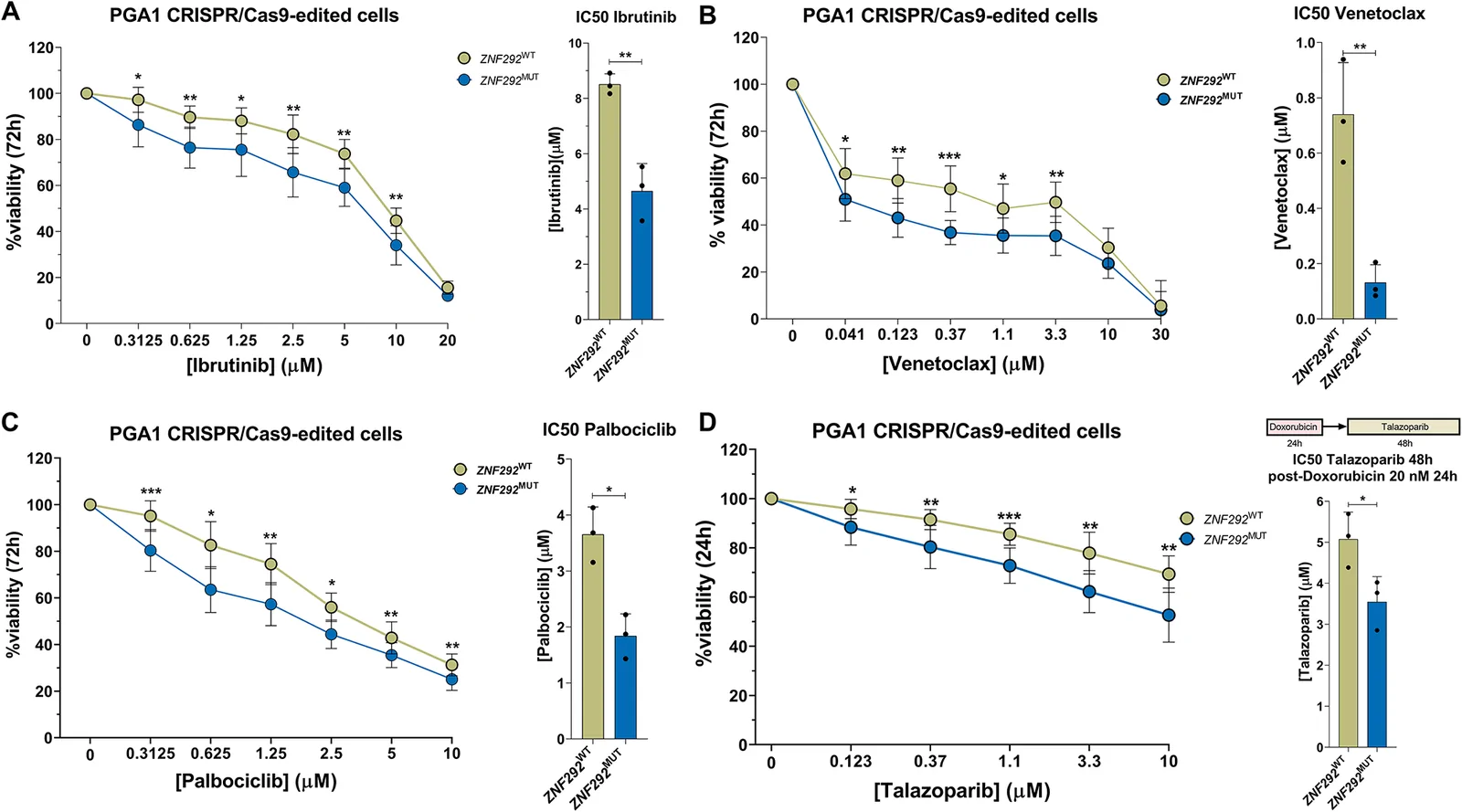
*ZNF292* mutations increase sensitivity to ibrutinib, venetoclax and palbociclib. **A**, **B**, **C**. Evaluation of cellular viability in the PGA1 CRISPR/Cas9-edited cells following 72 h of treatment with increasing concentrations of the BTK inhibitor ibrutinib (**A**), the BCL-2 inhibitor venetoclax (**B**) and the CDK4/6 inhibitor palbociclib (**C**). Left: dose-response survival curves relative to untreated. Data points represent mean ± SEM of 3 independent biological clones evaluated across 3 independent experiments. Two-Way ANOVA was performed. Right: half-maximal inhibitory concentrations (IC50), with points representing independent biological clones (n = 3). Two-tailed unpaired Student’s t-test was performed (* P < 0.05, ** P < 0.01, *** P < 0.001). **D**. Assessment of synthetic lethality targeting the DNA damage response in PGA1 CRISPR/Cas9-edited cells. Left: Dose-response viability curve following 24 h of treatment with the PARP inhibitor Talazoparib after 24 h of pretreatment with doxorubicin 20 nM (* P < 0.05, ** P < 0.01, *** P < 0.001). Curves represent mean ± SEM of 3 independent biological clones across 3 independent experiments, analyzed by Two-Way ANOVA. Right: IC50 bar chart. Data represent mean IC50 ± SD of 3 biological clones, compared using a two-tailed unpaired Student’s t-test (* P < 0.05)

To determine therapeutic vulnerabilities, we explored whether *ZNF292* mutations sensitize cells to frontline CLL therapies. Consistent with the transcriptional upregulation of BCR signaling observed in CRISPR/Cas9-edited cells and primary patient samples, *ZNF292* disruption conferred enhanced sensitivity to ibrutinib (Fig. 6A). Furthermore, *ZNF292*^MUT^ cells exhibited hypersensitivity to venetoclax, displaying a substantially lower IC50 (0.12 µM vs. 0.78 µM, Fig. 6B). These vulnerabilities were corroborated in the HG3 isogenic cells (Supplementary Fig. 12A-B). Additionally, given the dysregulation of cell cycle dynamics associated with *ZNF292* mutations, we assessed the response to the CDK4/6 inhibitor palbociclib. *ZNF292*^MUT^ cells were significantly more sensitive to palbociclib, as evidenced by a lower IC50 compared to *ZNF292*^WT^ (IC50: 2.00 µM vs. 3.53 µM) (Fig. 6C), a finding validated in HG3 cells (Supplementary Fig. 12C). Mechanistically, palbociclib induced a prominent accumulation of *ZNF292*^MUT^ cells in the G1 phase, preventing entry into S phase and resulting in a concomitant reduction of the G2/M population (Supplementary Fig. 12D). Moreover, p27 upregulation in *ZNF292*^MUT^ cells was associated with an increased sensitivity to pharmacological SKP2 inhibition, consistent with a greater reliance on SKP2-mediated regulation of p27 (Supplementary Fig. 12E).

Finally, considering that *ZNF292* mutations impair the cellular response to replication stress, we investigated whether this defect could create synthetic lethal vulnerabilities. We first determined an impaired ability to sustain G2/M checkpoint activation under replication stress conditions. Exposure to subtoxic doses of doxorubicin, a DNA-damaging agent known to induce replication-associated stress [46], resulted in a significantly reduced G2/M fraction in *ZNF292*^MUT^ cells compared to *ZNF292*^WT^ counterparts (Supplementary Fig. 13A-B). Based on this observation, we hypothesized that *ZNF292*^MUT^ cells may display increased dependency on PARP-mediated DNA repair. Interestingly, when cells were pretreated with doxorubicin to force G2/M checkpoint activation, *ZNF292*^MUT^ cells exhibited increased sensitivity to talazoparib (Fig. 6D, Supplementary Fig. 13C-D). These findings indicate that impaired replication stress response in *ZNF292*^MUT^ cells translates into a vulnerability to PARP inhibition, supporting a potential synthetic lethal interaction within DNA damage response pathways.

## Discussion

Mutations in *ZNF292* represent a recurrent genetic lesion affecting the RNA processing pathway in CLL [3–5, 21, 47], but how they contribute to CLL pathogenesis is still poorly understood. In this study, we show the independent prognostic value of *ZNF292* alterations in early-stage CLL, and demonstrate that *ZNF292* disruption rewires CLL biology by dysregulating cell cycle dynamics, impairing RNA and protein biosynthesis and compromising replication stress response, expanding the current understanding of the genetic architecture of CLL and unveiling novel therapeutic vulnerabilities associated with this genotype.

Our comprehensive mutational analysis suggests that *ZNF292* may act as a tumor suppressor gene in CLL. The mutational landscape was dominated by truncating variants, pointing to a loss-of-function as the main pathogenic mechanism. This pattern aligns with previous NGS-based studies reporting a high proportion of truncating *ZNF292* mutations (~ 65%)^5,21^. Importantly, the recurrent p.T2053fs variant, previously reported in CLL patients [3, 5, 21, 24], alongside the vast majority of identified mutations, systematically occur upstream of zinc-finger domains ZF9-11, pointing to the abrogation of DNA-binding capacity [22] as the key pathogenetic mechanism underlying *ZNF292* inactivation in CLL. In addition, a *ZNF292* mutation was detected in a patient with a 6q deletion in the 6q14 region, suggesting potential biallelic inactivation of this gene in CLL. Furthermore, the genomic context of these mutations reveals a strong enrichment within the IGHV-UM subtype, as previously reported [5, 24], and a frequent co-occurrence with *NOTCH1* mutations. VAF analyses indicated that *ZNF292* mutations emerge as subclonal acquisitions within *NOTCH1*-mutated clones. Remarkably, *ZNF292* mutations shorten TTFT within the *NOTCH1*-mutated subgroup, suggesting potential cooperation of both events in disease evolution. These clinical findings provide a compelling avenue for future functional dissection.

Initial sequencing efforts in CLL linked *ZNF292* alterations with shorter TTFT [3]. Subsequently, a comprehensive study by Knisbacher et al. integrating genetic and epigenetic features in a cohort of 506 patients (20 *ZNF292*-mutated cases) identified *ZNF292* mutations among a restricted set of genetic alterations independently associated with shorter OS and FFS [5]. However, their clinical relevance in early-stage disease, where risk stratification remains challenging due to the limited ability of current biomarkers to identify patients at risk of progression [48], has remained poorly characterized. This study corroborates the independent prognostic impact of *ZNF292* alterations, supporting their association with a clinically more aggressive disease with shorter TTFT and OS. Of note, we provide evidence that *ZNF292* alterations refine risk stratification within the Rai 0–1 subgroup. Importantly, multivariate survival analysis confirmed that *ZNF292* alterations retained their independent association with shorter TTFT even after accounting for the most commonly co-occurring mutations. These findings position *ZNF292* alterations as a clinically relevant prognostic factor in early-stage CLL. While our data highlight the association of *ZNF292* alterations with inferior survival in patients managed with diverse treatment strategies reflecting evolving clinical algorithms over the past two decades, their relevance in the era of targeted treatments warrants further reevaluation.

Beyond its clinical impact, this work provides the first mechanistic framework linking *ZNF292* deficiency to CLL pathogenesis through the dysregulation of global biosynthetic capacity, cell cycle dynamics and RS response. Because PGA1 and HG3 carry inherent limitations common to in vitro CLL models, most notably EBV-positive status, and our reliance on engineered biallelic knockout models whereas *ZNF292* mutations in patients are predominantly heterozygous, we complemented transcriptomic profiling and functional assays in CRISPR/Cas9-edited isogenic cells with primary patient RNA-seq data, providing evidence that *ZNF292* mutations impair protein synthesis efficiency. While mRNA translation defects have been previously implicated in CLL, most notably through *RPS15* mutations in CLL [49, 50], altered proteostasis is increasingly recognized as impacted by several genomic drivers [5]. By identifying *ZNF292* as a modulator of protein biosynthesis, we highlight aberrant proteostasis as a relevant pathway of CLL pathobiology.

Our study also provides evidence for a modulating effect of ZNF292 on cell cycle dynamics in CLL. Cell cycle regulation emerged as one of the most prominently transcriptionally dysregulated pathways in both CRISPR/Cas9-edited models and CLL patients. Our functional assays corroborated these findings, manifested as an extended S-phase duration. This delayed progression through S-phase is a potential consequence of high RS, a phenomenon similarly reported in other genetic contexts, such as *FBXW7*-mutated cells [51]. Mechanistically, we observed an uncoupling of DNA damage sensing from checkpoint execution: despite harboring higher levels of RS and DNA damage, *ZNF292*^MUT^ cells fail to efficiently activate the ATR-CHK1 axis. This signaling failure is further exacerbated by a defect in transcriptional regulation. Global attenuation of transcription is an essential protective mechanism to minimize transcription-replication conflicts (TRCs), which are a major source of endogenous DNA double-strand breaks [52]. Strikingly, while *ZNF292*^WT^ cells effectively reduced nascent RNA synthesis upon RS, *ZNF292*^MUT^ cells failed to suppress transcription. This inability to halt RNA synthesis may create a highly permissive environment for TRCs.

These mechanistic insights have direct therapeutic implications. Transcriptomic signatures of upregulated BCR signaling and altered apoptotic priming were associated with heightened sensitivity to ibrutinib and venetoclax, respectively. Consequently, while acknowledging the translational limitations of cell lines, our study provides in vitro proof-of-concept that *ZNF292*-mutated CLL cellular models exhibit therapeutic vulnerabilities that align with current frontline BTK and BCL2 inhibitors, a finding that warrants further research in primary CLL samples. Beyond these vulnerabilities, the altered cell cycle dynamics of *ZNF292*^MUT^ cells can be therapeutically exploited using CDK4/6 inhibitors. Given that an extended S-phase similarly sensitizes *NOTCH1*-mutated cells to palbociclib [53], our findings reinforce the potential of CDK4/6 inhibition as a promising therapeutic strategy in CLL subsets harboring cell cycle defects. Moreover, defects in G2/M checkpoint activation suggest potential vulnerabilities that could be exploited through synthetic lethal strategies targeting DNA damage response pathways.

## Conclusions

In summary, our study establishes *ZNF292* alterations as an independent prognostic factor associated with adverse clinical outcomes in early-stage CLL. We demonstrate that *ZNF292* mutations result in defective mRNA translation and modulate cell cycle dynamics, ultimately compromising the cellular response to replication stress. Moreover, our functional studies provide in vitro evidence that *ZNF292*-mutated cells exhibit increased sensitivity to ibrutinib and venetoclax, and identify additional pathways that may be exploitable for targeted therapeutic intervention.

## Supplementary Information

Below is the link to the electronic supplementary material.

Supplementary Material 1

Supplementary Material 2

Supplementary Material 3

## Data Availability

RNA-seq data are deposited at Gene Expression Omnibus (GEO) under accession number GSE329580. NGS datasets generated during the current study are available in the Sequence Read Archive (SRA) repository [BioProject ID: PRJNA1530468].

## Abbreviations

APC: Anaphase-promoting complex
ATR: Ataxia telangiectasia and Rad3-related
BCL2: B-cell lymphoma 2
BCR: B-cell receptor
BTK: Bruton’s tyrosine kinase
C2H2: Cys2His2 zinc-finger domain
CDK4/6: Cyclin-dependent kinase 4/6
CHK1: Checkpoint kinase 1
CI: Confidence interval
CLL: Chronic lymphocytic leukemia
CPT: Camptothecin
CRISPR/Cas9: Clustered regularly interspaced short palindromic repeats/CRISPR-associated protein 9
DAPI: 4′,6-diamidino-2-phenylindole
DEG: Differentially expressed gene
del(11q): Chromosome 11q deletion
del(13q): Chromosome 13q deletion
del(6q): Chromosome 6q deletion
EBV: Epstein-barr virus
EdU: 5-ethynyl-2’-deoxyuridine
EU: 5-ethynyl uridine
FBS: Fetal bovine serum
FDR: False discovery rate
FFS: Failure-free survival
GEO: Gene expression omnibus
GFP: Green fluorescent protein
GSEA: Gene set enrichment analysis
HR: Hazard ratio
IC50: Half-maximal inhibitory concentration
IGHV: Immunoglobulin heavy chain variable region
IGHV-UM: Unmutated immunoglobulin heavy chain variable region
iwCLL: international workshop on chronic lymphocytic leukemia
MACS: Magnetically activated cell sorting
MET: Mesenchymal-epithelial transition factor
mRNA: messenger RNA
MUT: Mutated
NES: Normalized enrichment score
NGS: Next-generation sequencing
OS: Overall survival
PARP: Poly (ADP-ribose) polymerase
PBS: Phosphate-buffered saline
PI: Propidium iodide
pRPA32: phosphorylated replication protein A 32 kDa subunit
RNA-seq: RNA sequencing
RPA32: Replication protein A 32 kDa subunit
RS: Replication stress
SD: Standard deviation
sgRNA: single guide RNA
SKP2: S-phase kinase-associated protein 2
SRA: Sequence read archive
SUnSET: Surface sensing of translation
TOP1: Topoisomerase I
TRC: Transcription-replication conflict
TTFT: Time to first treatment
VAF: Variant allele frequency
WT: Wild-type
ZNF292: Zinc-finger protein 292
γH2AX: Phosphorylated H2A histone family member X
