# Supplementary material for: ZNF292 mutations impair RNA and protein biosynthesis defining an adverse prognostic subset in early-stage chronic lymphocytic leukemia
Source: Biomark Res. 2026 Oct 1;14:130. doi: 10.1186/s40364-026-01005-y (PMC13632286; doi:10.1186/s40364-026-01005-y)
Supplement: Supplementary file 1 [file 40364_2026_1005_MOESM1_ESM.docx]

**SUPPLEMENTARY MATERIAL**

***ZNF292* mutations impair RNA and protein biosynthesis defining an adverse prognostic subset in early-stage chronic lymphocytic leukemia**

Alberto Rodríguez-Sánchez, María Alonso-Gago, Claudia Pérez-Carretero, Luis A. Corchete-Sánchez, Cristina Miguel-García, Sandra Santos-Minguez, Ángela Villaverde-Ramiro, Josefina Galende, Araceli Rubio, Rocío Benito-Sánchez, Federico Lo Schiavo, Martina Ghetti, Giorgia Simonetti, Ana E. Rodríguez-Vicente, Jesús M. Hernández Rivas* and Miguel Quijada-Álamo*

This section includes:

[**SUPPLEMENTARY METHODS**	2](#_Toc224812607)

[**SUPPLEMENTARY FIGURES**	15](#_Toc224812608)

[**SUPPLEMENTARY TABLES**	28](#_Toc224812609)

Supplementary Tables S8, S9 and S10 are indexed as .xls tables

# **SUPPLEMENTARY METHODS**

**Next-Generation Sequencing (NGS)**

Genomic DNA was isolated from CD19-positive B lymphocytes obtained from peripheral blood or bone marrow of CLL patients. These cells were isolated by magnetically activated cell sorting (MACS) using CD19 MicroBeads (Miltenyi Biotec, Bergisch Gladbach, Germany) following the manufacturer’s instructions. DNA extraction was performed using the AllPrep DNA/RNA Mini Kit (Qiagen #80284, Valencia, CA, USA) and the DNA was preserved at -80 ºC in RLT plus buffer. Quantitative and qualitative assessments of the genomic DNA were conducted employing the Qubit dsDNA HS Assay kit (Invitrogen, Life Technologies, Carlsbad, CA, USA) and the TapeStation 4200 system (Agilent Technologies, Santa Clara, CA, USA). The required concentration and quality to ensure optimal sequencing was 25 ng/µl and a DNA Integrity Number (DIN) > 6 respectively (as specified in the Illumina/Agilent protocols).

The Agilent SureSelect^QXT^ Target Enrichment system for Illumina Multiplexed Sequencing (Agilent Technologies) was employed to generate libraries of exonic regions from 54 CLL-related genes, encompassing 682 regions. This panel was previously validated^1–3^. The library contains oligonucleotide probes designed to align with *Homo sapiens* reference genomes, specifically hg19 GRCh37 (for panel version v1) and hg38 GRCh38 (for panel version v2). Targeted DNA sequencing libraries were constructed from 50 ng of genomic DNA using the SureSelect^QXT^ Library Prep Kit (G9681B) following manufacturer’s instructions. Tumor DNA was enzymatically fragmented and adapter-tagged to create libraries. Hybridization was performed using biotin-labeled probes specific to the target regions, followed by enrichment employing streptavidin beads. Subsequently, the libraries were amplified, dual-indexed, and pooled for sequencing. The quality of these libraries was evaluated using the 4200 TapeStation (Agilent Technologies). Library quantification was conducted using the Qubit 4.0 (Thermo Fisher Scientific, Waltham, MA, USA).

To verify mutations detected at low frequencies, PCR amplification-based techniques were employed. For validations, PCR libraries were generated using the Nextera XT DNA Sample Preparation Kit (Illumina, San Diego, CA, USA) and indexed using the Nextera XT Index Kit (Illumina). Following quality assessment on a Bioanalyzer DNA 1000 chip (Agilent Technologies) and purification using Agencourt AMPure XP beads (Beckman Coulter, Brea, CA, USA), the indexed libraries were quantified by fluorometry using the Qubit HS dsDNA assay kit (Invitrogen). The libraries were then combined for sequencing after being diluted to an equimolar concentration of 4 nM. Prior to denaturing and sequencing, a fluorometric measurement was used as a final validation of the concentration of the pooled library. Sequencing of the pooled genomic libraries was executed on the Illumina NextSeq or MiSeq platform (Ilumina).

To perform raw data quality control, FastQC v0.11.8 (Babraham Bioinformatics, Cambridge, UK) and Picard tools v2.2.4 (Broad Institute, Cambridge, MA, USA) were used to compile sequencing metrics. Demultiplexed files (FASTQ) were aligned to the reference genome, followed by identification of read duplicates using SAMTools (v1.3.1). Post-alignment processing was carried out with GATK v3.5 (Broad Institute), and coverage analysis for each targeted region was evaluated using BEDTools v.2.26.0. The threshold for high-quality sequencing results was set at a minimum quality score of Q30. Finally, an in-house pipeline integrating VarScan v2.4 (Washington University, St. Louis, MO, USA) for somatic variant calling and ANNOVAR v.2017Jul16 (Center for Applied Genomics, Philadelphia, PA, USA) for annotation was employed for comprehensive variant analysis.

Target regions achieved a median coverage of 600 reads/base, with at least 100X in 97% of them. To confirm variants found with VAF<5% detected in the custom panel, samples were resequenced using the amplicon-based techniques from Illumina Nextera XT, achieving read depths over 1000X. This high coverage allowed for the detection of variants down to 2%, as previously demonstrated by our group^1,2,4^.

Mutations were filtered based on their predicted impact, considering variants in the splice sites, untranslated regions (UTRs) and those leading to alterations in the amino acid sequence of the protein (missense, nonsense, and frameshift). Minor allelic frequencies (MAFs) were checked in different databases (dbSNP, 1000 genomes, ExAC, and our in-house database) to exclude single nucleotide polymorphisms (SNPs), with only variations having a MAF<0.01 selected for further investigation. In addition, variants exhibiting a VAF between 40-60% or greater than 90% were manually reviewed. Priority was given to those variants predicted to be deleterious, damaging, pathogenic or likely pathogenic according to *in silico* prediction tools (PolyPhen-2 (Polymorphism Phenotyping v2), SIFT (Sorting Intolerant From Tolerant) and ClinVar).

Variants were annotated using automated workflows, allowing the identification of potential pathogenic variants. Manual review using the Integrated Genomics Viewer (IGV) further validated the variants^5^. Additionally, ClinVar and the Varsome online tool were used to categorize the variations and assess their pathogenicity^6^.

**Cytogenetic and Fluorescence In Situ Hybridization (FISH) analysis**

Interphase FISH was performed on untreated peripheral blood or bone marrow samples using a standard four-probe CLL panel comprising *ATM*, CEP12, D13S319/13q34, and *TP53* (Vysis, Abbott Laboratories, Abbott Park, IL, USA). FISH processing and signal evaluations were carried out as previously described^7^. A minimum of 200 interphase nuclei with well-delineated signals were scored per sample. Cutoff thresholds for positivity were established at >10% for the remaining probes, based on internal laboratory cutoffs. Additionally, conventional chromosome banding analysis (G-banding cytogenetics) was performed to evaluate the presence of del(6q)^8^.

**Cell lines and culture conditions**

The CLL-derived HG3 and PGA1 cell lines were purchased from DMSZ (Deutsche Sammlung von Mikroorganismen and Zellkulturen) and incubated at 37ºC with 5% CO_2_ atmosphere. Cells were cultured in RPMI 1640 medium (Life Technologies, Paisley, UK) supplemented with 10% Fetal Bovine Serum (FBS, Life Technologies), 1% glutaMAX (Life Technologies) and 1% penicillin/streptomycin (Life Technologies). The identity and mutational status of the cell line were verified through NGS analysis in our customized panel. All cell lines used in this study were routinely tested for mycoplasma contamination to ensure experimental reliability. Mycoplasma detection was performed using the MycoAlert™ Mycoplasma Detection Kit (Lonza, Basel, Switzerland; LT07-318), according to the manufacturer’s instructions.

**Drugs and reagents**

Therapeutic agents and chemical inhibitors including venetoclax, palbociclib, SKP2 inhibitor C1 (SKPin C1), RO-3306 and AZD6738 were purchased from Selleckchem (Houston, TX, USA). Ibrutinib was obtained from LC laboratories (Woburn, MA, USA); doxorubicin from Sigma-Aldrich (St. Louis, MO, USA); Camptothecin (CPT) from Abcam (Cambridge, UK); and Talazoparib from Pfizer Oncology (New York, NY, USA). All compounds were reconstituted in DMSO obtained from Sigma for experimental use.

**CRISPR/Cas9 genome editing**

The PGA1 and HG3 cell lines, expressing constitutively Cas9 nuclease, were previously established and verified for functional Cas9 activity^9^. Single guide RNAs (sgRNAs) targeting the specific *loci* of *ZNF292* gene were selected based on predicted on-target efficiency scores and minimized off-target effects. As a negative control, we designed a sgRNA sequence that does not target the human genome. Detailed sequences are provided in Supplementary Table S4. Subsequently, pLKO5 vectors carrying the respective sgRNAs, were transfected into HG3-Cas9 and PGA1-Cas9 cells using the Amaxa Nucleofector II system (Lonza, Basel, Switzerland, program T-016 for HG3; program X-005 for PGA1). 48 hours following transfection, GFP-positive cells were isolated via sorting with a FACSAria II flow cytometer (BD Biosciences, Franklin Lakes, NJ, USA). Sorted cells were seeded and single-cell clones were expanded and screened. At least three different clones per condition were selected for the functional studies.

**Sequencing of sgRNAs targeting sites**

Genomic DNA isolation was performed using the QIAampDNA Micro Kit (Qiagen, Hilden, Germany) according to manufacturer’s instructions. PCR primers were designed using the Primer Blast tool available from the National Center for Biotechnology information (NCBI) (<https://www.ncbi.nlm.nih.gov/tools/primer-blast/>) to amplify the genomic regions flanking the upstream and downstream sgRNA cleavage sites for each mutation (Supplementary Table S4). Purification of PCR products was carried out for the screening of loss-of-function mutations using the High Pure PCR Product Purification Kit (Roche, Mannheim, Germany). Any resulting indels were verified by Sanger sequencing to confirm their presence at the expected genomic sites. The editing efficiency and allelic composition of each sgRNA were assessed using the Tracking of Indels by Decomposition (TIDE) software (<https://tide-calculator.nki.nl>; Netherlands Cancer Institute). Four independent *ZNF292*^MUT^ clones harboring frameshift mutations in *ZNF292* were selected from the PGA1 cell line. Similarly, three *ZNF292*^MUT^ clones were established in the HG3 cell line. These validated clones were expanded and utilized for all subsequent downstream analyses.

**RNA-seq data analysis**

RNA-sequencing was performed on independent clones derived from both the PGA1 (*ZNF292*^WT^ (n=4), *ZNF292*^MUT^ (n=4)) and HG3 (*ZNF292*^WT^ (n=3), *ZNF292*^MUT^ (n=3)) cell lines using the TruSeq Stranded mRNA protocol (Illumina). Briefly, the RNA library was prepared using 200 ng of total RNA from each sample and mRNAs were purified using poly-T oligo-attached magnetic beads and subsequently followed a fragmentation step. mRNA fragments were used as templates for first-strand cDNA synthesis by reverse transcription with random hexamers. Upon second-strand cDNA synthesis, double-stranded cDNAs were end-repaired and adenylated at the 3′ ends. Universal adapters were ligated to the cDNA fragments, then the sequencing library of DNA fragments that had adapters on both ends was amplified by PCR and used to produce the clusters that were sequenced in a NextSeq 550 platform (Illumina). Each sample was sequenced in a separate flow cell lane, with a final length of 76 bases.

Raw paired-end RNA-seq reads were processed with fastp (v0.23) to perform quality trimming and adapter removal, using stringent parameters to remove bases from both ends (-f 10, -F 10), discard reads shorter than 50 nucleotides (-l > 50), and filter known adapter sequences^10^. Transcript quantification was conducted within the RaNA-Seq^11^ analysis platform using Salmon to obtain transcript and gene expression estimates suitable for downstream differential analysis^12^.  Differential expression analysis was performed with the edgeR package^13^. Genes with adjusted p-value < 0.05 were considered as significant. Differential expression results were visualized and explored interactively using Rvisdiff^14^. Finally, functional pathway enrichment analysis was evaluated using the Gene Set Enrichment Analysis (GSEA) Preranked tool (Broad Institute). The entire transcriptomic dataset was ranked using the edgeR Likelihood Ratio (LR) statistic weighted by the direction of the change, calculated as sign (log_2_ foldchange) x LR. Enrichment was assessed against the Molecular Signatures Database (MSigDB) Reactome collections, defining statistical significance at a P < 0.05.

**RNA-seq data of CLLmap cohort**

Publicly available transcriptomic data were obtained from a previously published independent cohort^15^ (n=603) (Supplementary Table S9).

RNA-seq data from the CLLmap cohort were generated using stranded polyA-selected bulk RNA sequencing based on an automated implementation of the Illumina TruSeq Stranded mRNA library preparation protocol. Briefly, 200 ng of total RNA per sample underwent poly-T–based mRNA enrichment, heat fragmentation, and cDNA synthesis, followed by dual-indexed adapter ligation and PCR enrichment. Libraries were sequenced on Illumina short-read platforms (NovaSeq 6000, HiSeq 2000, or HiSeq 2500) using paired-end reads. Raw sequencing data were processed through the Broad Institute pipeline, including demultiplexing, quality control, and expression quantification. Reads were aligned using the STAR aligner to the hg19 (b37) reference genome with GENCODE v19 annotation, and gene-level expression matrices were generated using RNA-SeQC. To account for technical variability across centers and protocols, extensive batch-effect correction was applied using a linear modeling framework incorporating sequencing quality metrics, inferred technical covariates (including PEER factors), sequencing batch and center information, and additional preprocessing metrics. Batch-corrected log-transformed TPM expression values were used for gene set enrichment analyses (GSEA), defining statistical significance at a p< 0.05.

More details are in Knisbacher *et al*. *Nat Genet*, 2022^15^.

To evaluate the clinical impact of *ZNF292* expression levels, the optimal cutpoint for stratifying patients into high and low expression cohorts was determined using maximally selected rank statistics (the maxstat R package).

**Western blot analysis**

Cells were lysed for 30 minutes on ice with lysis buffer containing 140 mmol/l NaCl, 50 mmol/l EDTA, 10% glycerol, 1% Nonidet P-40, 20 mmol/l TrisHCl pH 7, supplemented with protease (cOmplete^TM^, Roche) and phosphatase inhibitors (PhosSTOP^TM^, Roche). Following centrifugation at 4ºC and 13.200 rpm for 5 minutes, the supernatant containing solubilized proteins was collected. Protein concentration was determined using Pierce™ BCA Protein Assay (Thermo Fisher, Bleiswijk, Netherlands), and samples were loaded onto polyacrylamide gels for electrophoresis under denaturing conditions using sodium dodecyl sulfate (SDS PAGE). Proteins were then transferred onto nitrocellulose membranes (GE Healthcare, Buckinghamshire, UK) and incubated with anti-human antibodies at 1:1 000 dilution. Protein expression levels were evaluated using anti-human antibodies. The following primary antibodies were purchased from Cell Signaling Technologies (Danvers, MA, USA): phospho-CHK1 (Ser345) (#2348, rabbit), CHK1 (#2360, mouse), γH2AX (#9718, rabbit), p27 (#3686, rabbit), SKP2 (#2652, rabbit), c-Myc/N-Myc (#13987, rabbit), anti-β-actin (#4967, Rabbit), anti-GAPDH (#5174, Rabbit) and anti α/β-tubulin (#2148, Rabbit). Anti-ZNF292 was purchased from FortisLife Science (Waltham, MA, USA; #A303-267A, Rabbit). Fluorescent secondary antibodies, including Goat anti-Rabbit IgG DyLight™ 800 (#35571) and Goat anti-Mouse IgG DyLight™ 680 (#35518), were acquired from Invitrogen. Immunoreactive bands were visualized, and near-infrared fluorescent signals were acquired using the Odyssey Imaging System (LI-COR Biosciences, Lincoln, NE, USA). Densitometric quantification of protein expression was performed using ImageJ (Fiji) software. The signal intensity of target proteins was normalized to loading control. All densitometric analyses were performed in independent duplicates, and data are expressed as relative protein levels between the protein of interest and the loading control.

**Cell cycle analysis**

Cellular DNA content and cell cycle progression were evaluated by flow cytometry. Briefly, 5 x 10^5^ exponentially growing *ZNF292*^WT^ and *ZNF292*^MUT^ cells were harvested, washed twice with cold PBS, and fixed dropwise in ice-cold 70% ethanol for at least 24 hours at -20°C. Fixed cells were subsequently washed and resuspended in a staining buffer containing Propidium Iodide (PI) and RNase A to ensure stoichiometric DNA staining, followed by a 30-minute incubation at room temperature in the dark. Samples were acquired on a BD Accuri C6 Plus flow cytometer (BD Biosciences). Cell cycle phase distribution (G0/G1, S, and G2/M fractions) was modeled and quantified using FlowJo software (FlowJo LLC, Ashland, OR, USA). Experiments were performed in at least 3 clones per genotype, with each clone analyzed in independent biological triplicates.

**G2/M Synchronization and Release Assay**

To evaluate mitotic progression, cells were synchronized at the G2/M boundary using the selective CDK1 inhibitor RO3306. Briefly, *ZNF292*^WT^ and *ZNF292*^MUT^ cells were seeded at a density of 1 × 10^6^ cells/mL and treated with RO-3306 9 µM for 20 hours under standard culture conditions. Following synchronization, cells were washed twice with pre-warmed PBS to effectively remove the inhibitor and released into fresh, pre-warmed complete culture medium. Cells were subsequently harvested at designated time points (0, 3, and 5 hours post-release) and analyzed by flow cytometry after DNA staining with propidium iodide. Experiments were performed in 3 clones per genotype, with each clone analyzed in independent biological triplicates.

**Cell viability analysis**

Longitudinal cell viability in response to drug exposure was assessed using the RealTime-Glo™ MT Cell Viability Assay (Promega, Madison, WI, USA) according to the manufacturer’s protocols. Briefly, *ZNF292*^WT^ and *ZNF292*^MUT^ cells were seeded in 96-well opaque white microplates at a density of 10 000 cells/well. The non-lytic luminescent reagents (MT Cell Viability Substrate and NanoLuc® Enzyme) were added concurrently with the drug treatments. Luminescence, which is directly proportional to the number of metabolically active cells, was continuously monitored over a 72-hour period using a Glomax microplate luminometer (Promega). Viability was calculated by normalizing the luminescent signal of treated cells to their respective vehicle-treated controls. All experiments were performed in technical triplicates, using 3 clones per genotype.

**Live-Cell Kinetic Proliferation Assay**

Real-time continuous monitoring of cell proliferation was performed using the IncuCyte® Live-Cell Analysis System (Sartorius, Göttingen, Germany). Given the suspension nature of the CLL cell lines, 96-well flat-bottom microplates were pre-coated with Poly-L-ornithine solution (Sigma-Aldrich) to facilitate stable cell adherence prior to seeding. Subsequently, *ZNF292*^WT^ and *ZNF292*^MUT^ clones were seeded in 96-well flat-bottom microplates at a density of 15 000 cells/well. Plates were placed within the IncuCyte incubator system maintained at 37°C and 5% CO2. Phase-contrast images were automatically acquired using a 20X objective at 6-hour intervals over a total period of 72 hours. Cellular proliferation kinetics were quantified by measuring the phase-object confluence (percentage of well area covered by cells) using the integrated IncuCyte Base Analysis Software. To account for minor variations in initial seeding, proliferation data were normalized to the time 0h measurement for each respective well. All conditions were evaluated in 4 biological clones per genotype in 2 independent experiments.

**Transwell Migration assay**

The migratory capacity of *ZNF292*^WT^ and *ZNF292*^MUT^ cells was evaluated using Transwell permeable supports with a 5 µm pore size polycarbonate membrane (Sarstedt, Nümbrecht, Germany). Prior to the assay, cells were serum-starved for 12 hours to reduce background signaling. Subsequently, 1 x 10^6^ cells resuspended in 100 µL of serum-free medium were seeded into the upper chamber of the Transwell insert. The lower chamber was filled with 700 µL of medium supplemented with 20% Fetal Bovine Serum to act as the chemoattractant. Spontaneous migration was assessed in parallel using serum-free medium in the lower chamber as a basal control. Following a 24-hour incubation at 37°C and 5% CO2, the inserts were carefully removed. The migrated cells in the lower chamber were collected and absolutely quantified using flow cytometry with BD Accuri C6+. Results were expressed as a migration index, defined as the fold-change of *ZNF292*^MUT^ cells migrated toward the chemoattractant relative to *ZNF292*^WT^ cells migration. Assays were performed in 3 clones per genotype in two independent experiments.

**Immunofluorescence and Confocal Microscopy**

To assess the basal and drug-induced accumulation of replication stress markers, *ZNF292*^WT^ and *ZNF292*^MUT^ cells were processed for immunofluorescence. Glass coverslips were pre-coated with a Poly-L-lysine solution (Sigma-Aldrich) to promote firm cellular adherence. Approximately 5 × 10^5^ cells were seeded onto the coated coverslips and allowed to attach for 60 minutes at 37°C. Following stable attachment, cells were gently washed and immediately fixed with 4% paraformaldehyde (PFA) for 15 minutes at room temperature, followed by permeabilization with 0.1% Triton X-100 in PBS for 10 minutes. After a 1-hour blockade in 5% Bovine Serum Albumin (BSA), coverslips were incubated overnight at 4°C with primary antibodies against phospho-RPA32 (#54762, rabbit, Cell Signaling Technologies). Following washing, cells were incubated with highly cross-adsorbed Alexa Fluor-conjugated secondary antibodies (Alexa Fluor 594 (Invitrogen)) for 1 hour at room temperature in the dark. Nuclei were counterstained with DAPI, and coverslips were mounted onto glass slides using VectaShield. High-resolution images were acquired using a Leica SP8 laser scanning confocal microscope equipped with a 63X oil-immersion objective. Quantitative image analysis, including the scoring of nuclear foci per cell, was performed using ImageJ (Fiji) software on at least 100 cells randomly selected cells per condition, with 3 *ZNF292*^WT^ and *ZNF292*^MUT^ clones across 2 independent replicates.

**REFERENCES**

1.	Pérez-Carretero C, Hernández-Sánchez M, González T, Quijada-Álamo M, Martín-Izquierdo M, Santos-Mínguez S, et al. TRAF3 alterations are frequent in del-3’IGH chronic lymphocytic leukemia patients and define a specific subgroup with adverse clinical features. Am J Hematol. 2022 Jul;97(7):903–14.

2.	Pérez-Carretero C, Hernández-Sánchez M, González T, Quijada-Álamo M, Martín-Izquierdo M, Hernández-Sánchez JM, et al. Chronic lymphocytic leukemia patients with IGH translocations are characterized by a distinct genetic landscape with prognostic implications. Int J Cancer. 2020;147(10):2780–92.

3.	Hernández-Sánchez M, Rodríguez-Vicente AE, González-Gascón y Marín I, Quijada-Álamo M, Hernández-Sánchez JM, Martín-Izquierdo M, et al. DNA damage response-related alterations define the genetic background of patients with chronic lymphocytic leukemia and chromosomal gains. Exp Hematol. 2019;72:9–13.

4.	Quijada-Álamo M, Hernández-Sánchez M, Robledo C, Hernández-Sánchez JM, Benito R, Montaño A, et al. Next-generation sequencing and FISH studies reveal the appearance of gene mutations and chromosomal abnormalities in hematopoietic progenitors in chronic lymphocytic leukemia. J Hematol Oncol. 2017;10(1):1–11.

5.	Robinson JT, Thorvaldsdóttir H, Wenger AM, Zehir A, Mesirov JP. Variant review with the integrative genomics viewer. Cancer Res. 2017;77(21):e31–4.

6.	Kopanos C, Tsiolkas V, Kouris A, Chapple CE, Albarca Aguilera M, Meyer R, et al. VarSome: the human genomic variant search engine. Bioinformatics. 2019;35(11):1978–80.

7.	González MB, Hernández JM, García JL, Lumbreras E, Castellanos M, Hernández JM, et al. The value of flourescence in situ hybridization for the detection of 11q in multiple myeloma. Haematologica. 2004;89(10):1213–8.

8.	Pérez Carretero C, González T, Quijada Álamo M, Rigolin GM, Dubuc A, Villaverde Ramiro Á, et al. Chronic lymphocytic leukemia patients with chromosome 6q deletion as the sole cytogenetic abnormality display a high frequency of RPS15 mutations and have a poor prognosis. Am J Hematol. 2024;99(9):1845–8.

9.	Quijada-Álamo M, Hernández-Sánchez M, Alonso-Pérez V, Rodríguez-Vicente AE, García-Tuñón I, Martín-Izquierdo M, et al. CRISPR/Cas9-generated models uncover therapeutic vulnerabilities of del(11q) CLL cells to dual BCR and PARP inhibition. Leukemia. 2020;34(6):1599–612.

10.	Chen S, Zhou Y, Chen Y, Gu J. Fastp: An ultra-fast all-in-one FASTQ preprocessor. Bioinformatics. 2018;34(17):i884–90.

11.	Prieto C, Barrios D. RaNA-Seq: Interactive RNA-Seq analysis from FASTQ files to functional analysis. Bioinformatics. 2020;36(6):1955–6.

12.	Patro R, Duggal G, Love MI, Irizarry RA, Kingsford C. Salmon provides fast and bias-aware quantification of transcript expression. Nat Methods. 2017;14(4):417–9.

13.	Robinson MD, McCarthy DJ, Smyth GK. edgeR: A Bioconductor package for differential expression analysis of digital gene expression data. Bioinformatics. 2009;26(1):139–40.

14.	Barrios D, Prieto C. Rvisdiff: An R package for interactive visualization of differential expression. Front Bioinforma. 2024;4(September):1–5.

15.	Knisbacher BA, Lin Z, Hahn CK, Nadeu F, Duran-Ferrer M, Stevenson KE, et al. Molecular map of chronic lymphocytic leukemia and its impact on outcome. Nat Genet. 2022;54(11):1664–74.

# **SUPPLEMENTARY FIGURES**

**Supplementary Figure 1. Evolutionary conservation of *ZNF292* missense variants and mutational validation in the CLLmap cohort.**

**A.** Sequence alignment demonstrating the high evolutionary conservation of the specific amino acid residues affected by *ZNF292* missense mutations identified in the study cohort across diverse vertebrate species. **B.** Lollipop plot mapping the somatic *ZNF292* mutations identified in the CLLmap patient cohort, confirming the widespread distribution and the predominance of truncating alterations across the gene body. **C.** Scatter plot depicting the Cancer Cell Fraction (CCF, %) distribution of *ZNF292* mutations within the CLLmap cohort. **D.** *ZNF292* mRNA expression log_10_ (TPM + 1)) in *ZNF292*^WT^ vs. *ZNF292*^MUT^ patients from the CLLmap cohort. **E.** Percentage of patients harboring major cytogenetic alterations [del(13q), tri12, del(17p), del(11q)] in *ZNF292*^WT^ and *ZNF292*^MUT^ cases from the Salamanca cohort. * P < 0.05.

**Supplementary Figure 2. Clinical analyses in the study cohort.**

**A**. Bar plot showing the percentage of patients with *NOTCH1* mutations from the CLLmap stratified by *ZNF292* mutational status.

**B.** Scatter plot comparing the Variant Allele Frequencies (VAF, %) of co-occurring *ZNF292* and *NOTCH1* mutations within the same patients, illustrating their clonal hierarchy and subclonal distribution. The diagonal line represents a theoretical 1:1 VAF ratio.

**C-D.** Kaplan-Meier survival curves for Time to First Treatment (TTFT) (**C**) and Overall Survival (OS) (**D**) comparing *ZNF292*^WT^ patients, *ZNF292*^MUT^ patients, and a subset of patients harboring del(6q14).

**E-F.** Impact of *ZNF292* mutation type on (**E**) TTFT and (**F**) OS.

**G-H.** Prognostic impact of *ZNF292* mutations within early-stage disease. Kaplan-Meier curves demonstrate a significantly shorter median TTFT (**G**) and OS (**H**) in Binet A patients carrying *ZNF292* mutations compared to their unmutated counterparts.

**I.** Kaplan-Meier analysis for TTFT stratified by the mutational status of both *ZNF292* and *NOTCH1*, revealing the negative clinical impact when both genetic alterations co-occur.

**Supplementary Figure 3. Clinical analyses in the CLLmap cohort.**

**A–C.** Kaplan-Meier survival curves comparing *ZNF292*^WT^ and *ZNF292*^MUT^ patients across the entire international CLLmap cohort for Time to First Treatment (TTFT) **(A)**, Failure-Free Survival (FFS) **(B)**, and Overall Survival (OS) **(C)**.

**D-E.** Survival analysis restricted to the unmutated IGHV (IGHV-UM) patient subgroup. Kaplan-Meier curves demonstrate a significantly shorter OS **(D)** and FFS **(E)** for *ZNF292*^MUT^ patients.

**F.** Overall survival analysis specifically evaluating the treatment-naïve subset of IGHV-UM patients within the CLLmap cohort.

**G-H.** Prognostic impact of *ZNF292* mRNA expression levels. Kaplan-Meier curves for Overall Survival **(G)** and Failure-Free Survival **(H)** stratifying the CLLmap cohort into *ZNF292*-Low and *ZNF292*-High expression groups.

**I.** Kaplan-Meier OS analysis in the CLLmap cohort of patients treated with chemotherapy or chemoimmunotherapy.

**Supplementary Figure 4. Multivariate Cox regression analysis of TTFT and OS in the entire study cohort.**

Forest plots illustrating the independent prognostic impact of *ZNF292* alterations (mutations or del(6q)14) on Time to First Treatment (TTFT, top) and Overall Survival (OS, bottom). The analysis adjusts for clinical and biological risk factors, including *IGHV* status, Rai stage, and CLL driver mutations. Hazard ratios (HR) and 95% confidence intervals (CI) are displayed. Red indicators denote statistically significant covariates (P < 0.05).

**Supplementary Figure 5. Generation and validation of CRISPR/Cas9-edited cells.**

**A.** Representative Sanger sequencing chromatograms of the *ZNF292* target locus in the PGA1 cells. Alignments compare the wild-type (WT) control sequence with four independent CRISPR/Cas9-edited mutated cells (#4, #7, #12, and #15). Red boxes highlight the specific insertions and deletions (indels) introduced at the cleavage site, leading to frameshift mutations. **B.** Representative Sanger sequencing chromatograms validating the successful editing of the *ZNF292* locus in the HG3 cells. Distinct frameshift-inducing indels are detailed for mutant clones #9, #17, and #34 compared to the WT control.

**C.** Western blot analysis confirming the complete ablation of the ZNF292 protein in the generated isogenic models.

**Supplementary Figure 6. Transcriptomic profiling of *ZNF292*-mutated CRISPR/Cas9-edited cells**

**A.** Heatmap representing unsupervised hierarchical clustering of differentially expressed genes (FDR < 0.01; |fold change| > 0.75) between *ZNF292*^WT^ and CRISPR/Cas9-edited *ZNF292*^MUT^ clones in the PGA1 cells. Columns represent individual biological replicates, and rows represent individual genes. **B.** Sample correlation matrix and dendrogram demonstrating high inter-replicate reproducibility and distinct global clustering between *ZNF292*^WT^ and *ZNF292*^MUT^ cells of the PGA1 CRISPR/Cas9-edited cells. **C.** Volcano plot displaying the differential gene expression landscape in PGA1 isogenic cells. Red dots indicate significantly dysregulated genes according to the established statistical thresholds (-log_10_ p-value and logFC). **D-E.** Validation of transcriptomic alterations in the HG3 *ZNF292*-mutated cells, illustrated by a heatmap of differentially expressed genes (**D**) and a volcano plot (**E**).

**Supplementary Figure 7. Pathway enrichment analysis reveals consistent downregulation of cell cycle and DNA repair *ZNF292*-mutated CRISPR/Cas9-edited cells.**

**A.** Bar plots summarizing the Gene Set Enrichment Analysis (GSEA) results for the PGA1 isogenic cells. Significantly downregulated pathways (p-value < 0.05) in *ZNF292*^MUT^ cells compared to wild-type controls are grouped by biological categories: Cell cycle, DNA Damage Response (DDR), RNA metabolism, Apoptosis, Immune system, and DNA-related pathways. The x-axis represents the negative Normalized Enrichment Score (NES).

**B.** Pathway enrichment analysis of the Differentially Expressed Genes (DEGs; |log_2_ fold change| > 1 and FDR < 0.01) identified in the HG3 *ZNF292*^MUT^ cells. Bars indicate the significance (-log_10_ p-value) of the enriched biological processes, confirming the marked dysregulation of mRNA splicing, cell cycle checkpoints, translation, and DNA repair.

**C.** Venn diagram illustrating the intersection of significantly downregulated genes between the PGA1 and HG3 *ZNF292*-mutated isogenic cells. The specific set of commonly downregulated genes (*DDX39B*, *PCDHGC3*, *HLA-A*, *HLA-B*, and *HLA-C*) is highlighted in the overlapping region.

**Supplementary Figure 8. GSEA in the CLLmap cohort validates the dysregulation of cell cycle and translational pathways in *ZNF292*-mutated patients.**

**A.** Representative GSEA enrichment plots detailing the most significantly upregulated Reactome pathways in *ZNF292*^MUT^ patients compared to their *ZNF292*^WT^ counterparts. *ZNF292* disruptions are associated with a marked positive enrichment in networks driving B-cell receptor (BCR) regulation or Fc epsilon receptor (FCERI) signaling.

**B.** Representative GSEA enrichment plots illustrating significantly downregulated Reactome pathways in *ZNF292*^MUT^ patients. Consistent with the *in vitro* models, primary clinical samples exhibit a strong negative enrichment in pathways governing cell cycle progression (e.g., ubiquitin-dependent degradation of mitotic proteins, stabilization of p53) and global protein synthesis (eukaryotic translation initiation and elongation). Each plot displays the Normalized Enrichment Score (NES) and the corresponding nominal *P*-value.

**Supplementary Figure 9. Disruption of *ZNF292* significantly alters the expression of genes driving proteostasis, translation, and cell cycle progression in primary CLL patients.**

Scatter dot plots comparing the normalized mRNA expression levels (represented as log_10_ (TPM+1)) of selected genes between *ZNF292*^WT^ and *ZNF292*^MUT^ patients. The panels detail key components categorized by their biological function: Proteostasis and proteasome machinery (*PSMA7*, *PSMB3*, *PSMB7*, and *PSMD12*), showing consistent downregulation in mutated patients; Translation initiation and ribosomal function (*EIF2B1* and *RPL39L*), which are significantly repressed on *ZNF292*-mutated cases; Cell cycle regulation (*MYBL2* and *CCNE1*), highlighting a significant upregulation of the *MYBL2* transcription factor and a trend towards increased Cyclin E in the *ZNF292*-mutated subgroup; and BCR signaling (BTK, IGHV1-69, IGLV3-21, IGHM). Central horizontal lines represent the mean, and error bars denote the standard deviation. *P < 0.05, **P < 0.01.

**Supplementary Figure 10. *ZNF292* deficiency impairs cellular migration and extends S-phase duration.**

**A.** Cell cycle distribution analysis in the HG3 isogenic cells. Representative Propidium Iodide (PI) histograms (left) and corresponding quantitative bar graphs (right). Data are represented as mean ± SD; *P < 0.05, **P < 0.01.

**B.** Gene Set Enrichment Analysis (GSEA) enrichment plot demonstrating a negative enrichment (NES = -1.61) for the Reactome pathway regulating the "SCF-SKP2 mediated degradation of p27/p21" in *ZNF292*^MUT^ PGA1 cells.

**C.** Western blot validation of key cell cycle regulators. CRISPR/Cas9-mediated ablation of *ZNF292* results in a marked accumulation of p27 protein levels, alongside a concurrent downregulation of SKP2 and the cMyc transcription factor. Tubulin serves as the loading control.

**D.** Scatter plot illustrating a significant inverse correlation between the mRNA expression levels of *ZNF292* and *CDKN1B* (gene encoding p27) within the primary CLLmap patient cohort. The red line represents the linear regression fit.

**Supplementary Figure 11. *ZNF292* loss impairs the DNA Damage Response (DDR) and exacerbates DNA damage accumulation under replication stress.**

**A.** Flow cytometry analysis of apoptosis in the PGA1 CRISPR/Cas9-edited cells using Annexin V and Propidium Iodide (PI) double staining following treatment with the Topoisomerase I inhibitor camptothecin (CPT, 5 µM) for 24 hours.

**B.** Western blot evaluation of the DDR signaling cascade under basal conditions and upon replication stress (CPT 5 µM, 2h) in PGA1 isogenic cells. Left panels show representative blots for pCHK1, total CHK1, and γH2AX, with Tubulin and GAPDH serving as loading controls. Right bar graphs display the densitometric quantification of the pCHK1/CHK1 and γH2AX/GAPDH ratios.

**C.** Quantification of global nascent RNA synthesis via EU incorporation under replication stress. Scatter dot plots represent the integrated fluorescence density in individual *ZNF292*^WT^ and *ZNF292*^MUT^ PGA1 cells, untreated or exposed to 5 µM CPT for 2 hours.

**D.** Cell cycle profiling in response to pharmacological ATR inhibition. Representative PI histograms (left) and corresponding quantitative bar graphs (right) detailing the cell cycle phase distribution (G0/G1, S, and G2/M) of *ZNF292*^WT^ and *ZNF292*^MUT^ PGA1 cells after 48 hours of treatment with the ATR inhibitor AZD6738 (1 µM). Data are represented as mean ± SD; *P < 0.05, **P < 0.01, ***P < 0.001, ****P < 0.0001.

**Supplementary Figure 12. *ZNF292* deficiency confers selective vulnerability to targeted inhibition of BTK, BCL2, CDK4/6 and SKP2.**

**A-C.** Dose-response curves illustrating the cytotoxic effect of ibrutinib (**A**), venetoclax (**B**) and palbociclib (**C**) in HG3 isogenic cells. Cells were treated with increasing concentrations of each drug for 72 hours. Viability was calculated compared to untreated cells.

**D.** Cell cycle distribution analysis following pharmacological CDK4/6 inhibition. Representative Propidium Iodide (PI) flow cytometry histograms (**left**) and corresponding quantitative bar graphs (**right**) showing the percentage of cells in G0/G1, S, and G2/M phases after treatment with Palbociclib (4 µM for 72 hours).

**E.** Cell viability assessment in the PGA1 isogenic cells following 72 hours of treatment with a pharmacological SKP2 inhibitor.

Data are presented as mean ± SEM of independent experiments. *P < 0.05, **P < 0.01, ***P < 0.001.

**Supplementary Figure 13. *ZNF292* deficiency impairs the doxorubicin-induced G2/M checkpoint and sensitizes cells to PARP inhibition following replication stress.**

**A.** Dose-response viability curves of *ZNF292*^WT^ and *ZNF292*^MUT^ PGA1 CRISPR/Cas9-edited cells treated with the topoisomerase II inhibitor doxorubicin for 24 hours.

**B.** Cell cycle profiling following the induction of replication stress with doxorubicin in PGA1 CRISPR/Cas9-edited cells. Representative propidium iodide (PI) histograms (left) and corresponding quantitative bar graphs (right) of cells treated with 10 nM or 20 nM Doxorubicin for 24 hours.

**C.** Cell viability assessment of PGA1 isogenic cells after 72 hours of treatment with the PARP inhibitor Talazoparib as a single agent.

**D.** Sequential treatment viability assay. PGA1 isogenic cells were pre-treated with 20 nM Doxorubicin for 24 hours, followed by exposure to increasing concentrations of Talazoparib for 48 hours. Data are presented as mean ± SD of independent biological replicates. *P < 0.05, **P < 0.01.

# **SUPPLEMENTARY TABLES**

**Supplementary table S1: Clinical and biological characteristics of CLL patients**

**depending on *ZNF292* mutational status (N=418)**

| **Characteristic** | | ***ZNF292*^WT^ (n=393)** | ***ZNF292*^MUT^ (n=25)** | **P-value** |
| --- | --- | --- | --- | --- |
| **Median follow up, months** | | 74 | 63 | 0.44^b^ |
| **Median age at diagnosis** | | 71 (37 – 113) | 69 (25 – 87) | 0.257^b^ |
| **Gender male, %** | | 63.6 | 65.4 | 0.516^a^ |
| **del(13q), %** | | **49.4** | **29.2** | **0.042^a^** |
| **del(11q), %** | | 9.3 | 9.1 | 0.666^a^ |
| **tri12, %** | | 32.9 | 44.0 | 0.178^a^ |
| **del(17p), %** | | 14.4 | 13.6 | 0.607^a^ |
| **IGHV-UM, %** | | **50.8** | **76.0** | **0.012^a^** |
| **Binet stage** | **A, %** | 74.6 | 62.5 | 0.144^a^ |
|  | **B/C, %** | 25.4 | 37.5 |  |
| **Rai stage** | **0/I, %** | 78.8 | 79.2 | 0.602^a^ |
|  | **II/III/IV, %** | 21.2 | 20.8 |  |
| **Need for treatment, %** | | **56.1** | **76.9** | **0.028^a^** |
| **Lymphocytes (x10^9^/L), median (range)** | | 13.1 (1.2 – 212) | 10.1 (2.4 – 33.5) | 0.574^b^ |
| **White blood cells (x10^9^/L), median (range)** | | 18.1 (2.3 – 369) | 16.8 (5.6 – 39) | 0.530^b^ |
| **Platelet (x10^9^/L), median (range)** | | 189 (112 – 320) | 199 (17 – 272) | 0.669^b^ |
| **Hemoglobin (g/L), median (range)** | | 14.1 (4.4 – 18.9) | 14.4 (7.9 – 16.6) | 0. 671^b^ |
| **High β2 microglobulin level, %** | | 40.4 | 50.0 | 0.651^a^ |
| **High lactate dehydrogenase level, %** | | 20.7 | 31.8 | 0.165^a^ |
| **B symptoms, %** | | 8.6 | 15.0 | 0.260^a^ |
| **Adenopathies, %** | | 41.1 | 45.5 | 0.800^a^ |
| **Hepatomegaly, %** | | 5.0 | 0.0 | 0.531^a^ |
| **Splenomegaly, %** | | 18.7 | 16.7 | 0.936^a^ |

**a**: χ2 test

**b**: Mann-Whitney U test

*Samples were collected between 2004 and 2025.

**Supplementary Table S2: Treatment regimens and therapeutic strategies administered in the study cohort.**

**Supplementary Table S3: Coverage Metrics for Genomic Regions within the Custom NGS Panel of CLL-Related Genes**

| **Gene** | **Transcript** | **Regions** | **Mean coverage (reads/base)** |
| --- | --- | --- | --- |
| *ARID1A* | ENST00000324856 | Exons 1-20 | 814 |
| *ASXL1* | ENST00000375687 | Exons 1-13 | 701 |
| *ATM* | ENST00000278616 | Exons 2-63 | 456 |
| *ATRX* | ENST00000373344 | Exons 1-35 | 243 |
| *BAX* | ENST00000345358 | Exon 2-6 | 826 |
| *BAZ2A* | ENST00000551812 | Exons 2-28 | 819 |
| *BCL2* | ENST00000333681 | Exons 2-3 and 5'UTR | 536 |
| *BCOR* | ENST00000378444 | Exons 2-15 | 398 |
| *BIRC3* | ENST00000263464 | Exons 2-9 | 483 |
| *BRAF* | ENST00000288602 | Exons 11-16 | 499 |
| *BTK* | ENST00000308731 | Exons 2-19 | 456 |
| *CARD11* | ENST00000396946 | Exons 3-17 | 795 |
| *CCND2* | ENST00000261254 | Exons 1-5 | 827 |
| *CD19* | ENST00000324662 | Exons 1-6 | 845 |
| *CDC73* | ENST00000367435 | Exons 1-16 | 436 |
| *CHD2* | ENST00000394196 | Exons 2-39 | 463 |
| *DDX3X* | ENST00000399959 | Exons 1-16 | 376 |
| *EGR2* | ENST00000242480 | Exons 1-2 | 1021 |
| *FAM50A* | ENST00000393600 | Exons 2-12 | 478 |
| *FAT1* | ENST00000441802 | Exons 2-27 | 754 |
| *FBXW7* | ENST00000281708 | Exons 7-12 | 721 |
| *FUBP1* | ENST00000370768 | Exons 1-19 | 451 |
| *HIST1H1B* | ENST00000331442 | Exon 1 | 954 |
| *HIST1H1E* | ENST00000304218 | Exon 1 | 668 |
| *IGLL5* | ENST00000526893 | Exons 1-3, 5'UTR | 555 |
| *IKZF3* | ENST00000346872 | Exon 5 | 696 |
| *IRF4* | ENST00000380956 | Exons 2-9 | 698 |
| *KLHL6* | ENST00000341319 | Exons 1-7 | 693 |
| *KRAS* | ENST00000311936 | Exons 2-5 | 543 |
| *MAP2K1* | ENST00000307102 | Exons 1-11 | 561 |
| *MED12* | ENST00000374080 | Exons 1-4 | 389 |
| *MGA* | ENST00000570161 | Exons 2-23 | 572 |
| *MYD88* | ENST00000396334 | Exons 2-5 | 781 |
| *NFKBIE* | ENST00000275015 | Exons 1-2 | 969 |
| *NOTCH1* | ENST00000277541 | Exon 34 and 3'UTR | 1208 |
| *NRAS* | ENST00000369535 | Exons 2-3 | 587 |
| *NXF1* | ENST00000294172 | Exons 3-21 | 653 |
| *PAX5* | ENST00000358127 | enhancer | 791 |
| *PCDH10* | ENST00000264360 | Exons 1-5 | 562 |
| *PIK3CD* | ENST00000377346 | Exons 3-24 | 848 |
| *PLCG2* | ENST00000564138 | Exons 2-33 | 618 |
| *POT1* | ENST00000357628 | Exons 5-19 | 405 |
| *PTPN11* | ENST00000351677 | Exonso 2-15 | 645 |
| *RPS15* | ENST00000593052 | Exons 2-4 | 679 |
| *SAMHD1* | ENST00000262878 | Exons 1-16 | 465 |
| *SETD2* | ENST00000409792 | Exons 1-21 | 476 |
| *SF3B1* | ENST00000335508 | Exons 14-16 | 524 |
| *SORCS2* | ENST00000507866 | Exons 1-27 | 876 |
| *TP53* | ENST00000269305 | Exons 4-10 | 634 |
| *TRAF3* | ENST00000392745 | Exons 1-12 | 641 |
| *XPO1* | ENST00000401558 | Exons 15-16 | 556 |
| *ZC3H18* | ENST00000301011 | Exons 2-18 | 780 |
| *ZMYM3* | ENST00000373998 | Exons 2-25 | 507 |
| *ZNF292* | ENST00000339907 | Exons 1-8 | 401 |

**Supplementary table S4. Sequences of sgRNAs and primers for generation of CRISPR/Cas9-edited CLL cells**

| **Target** | **Forward (5’-3’)** | **Reverse (5’-3’)** |
| --- | --- | --- |
| ***ZNF292* exon8 sgRNA1** | CACCG**GGATCACTGCTACATTACTG** | AAAC**CAGTAATGTAGCAGTGATCC**C |
| ***ZNF292* exon8 sgRNA2** | CACCG**GCATAATACGCATCTACTGT** | AAAC**ACAGTAGATGCGTATTATGC**C |
| **Control sgRNA1** | CACCG**ACGGAGGCTAAGCGTCGCAA** | AAAC**TTGCGACGCTTAGCCTCCGT**C |
|  |  |  |
| **Primer 1 *ZNF292* sgRNA1** | GTCAGTTGGTGCATCATTTTACAAC | ACTCAAGGGATTGGGAAACTGG |
| **Primer 2 *ZNF292* sgRNA2** | TTTGCTTTTTCACAGACTGAAGG | ACAAGTAACAGCTCACAGCG |

**Supplementary Table S5. Associations and Mutational Co-occurrences in *ZNF292*-mutated Patients: Odds Ratios, 95% Confidence Intervals, and Nominal vs. Adjusted** $\boldsymbol{p}$**-values.**

**Supplementary Table S6: Impact of *ZNF292* mutations on clinical outcomes in IGHV-UM CLL patients from the CLLmap cohort (extracted from Knisbacher *et al*., Nat Genetics, 2022)**

**Supplementary Table S7. Bivariate Cox Proportional Hazards Analysis for TTFT and Overall Survival Adjusted for Individual Covariates**

**TTFT	OS**
